# Electrochemical sensor based on PEDOT/CNTs-graphene oxide for simultaneous determination of hazardous hydroquinone, catechol, and nitrite in real water samples

**DOI:** 10.1038/s41598-024-54683-9

**Published:** 2024-03-07

**Authors:** Yousef M. Ahmed, Mahmoud A. Eldin, Ahmed Galal, Nada F. Atta

**Affiliations:** Chemistry Department, Faculty of Science, Cairo University, Giza, 12613 Egypt

**Keywords:** Graphene oxide, Carbon nanotubes, Conducting polymer, Hydroquinone, Catechol, Nitrite, Analytical chemistry, Electrochemistry

## Abstract

Hydroquinone (HQ), catechol (CC) and nitrite (NT) are considered aquatic environmental pollutants. They are highly toxic, harm humans’ health, and damage the environment. Thus, in the present work we introduce a simple and efficient electrochemical sensor for determination of HQ, CC, and NT simultaneously in wastewater sample. The sensor is fabricated by modifying the surface of a glassy carbon electrode (GCE) by two successive thin films from poly(3,4-ethylenedioxythiophene) (PEDOT) and a mixture of carbon nanotubes-graphene oxide (CNT-GRO). Under optimized conditions the HQ, CC, and NT are successfully detected simultaneously in wastewater sample with changing their concentrations in the ranges (0.04 → 100 µM), (0.01 → 100 µM) and (0.05 → 120 µM), the detection limits are 8.5 nM, 3.8 nM and 6.1 nM, respectively. Good potential peak separations: 117 mV and 585 mV are obtained between the HQ-CC, and CC-NT. The sensor has an excellent catalytic capability toward the oxidation of HQ, CC, and NT due to good synergism between its composite components: PEDOT, GRO and CNTs. The features of the sensor are large active surface area, good electrical conductivity, perfect storage stability, good reproducibility, anti-interference capability and accepted recovery rate for HQ, CC, and NT determination in wastewater sample.

## Introduction

Phenolic compounds and nitrite (NT) are pollutant species existing in aquatic environment. Phenolic compounds such as hydroquinone (HQ) and catechol (CC) are always co-existed and widespread in different industries such as pharmaceuticals, cosmetics, leather, plastic, dyes, textiles, and pesticides. Wastes resulting from these industries can lead to environmental pollution^1^. United States environmental protection agency and European Union listed the two isomers HQ and CC as environmental pollutants^2^. These compounds are highly toxic and poorly degradable, thus they can both harm humans’ health and damage the environment^3–5^. It is of great interest in analytical field to detect simultaneously the two isomers: HQ and CC due to their similar structures and characteristics^6^. Nitrite (NT) is an environmental contaminant with high toxicity^7,8^. It is extensively used in industrial, food additive, fertilizing agents, and inhibitors^9–11^. High concentration of NT can cause diseases, such as “blue baby syndrome”, cancer, and hypertension^12,13^. Further, in the human body, nitrite can be converted into carcinogenic nitroso-compounds (nitroso-amide and nitroso-amine) which lead to the formation of tumors in stomach, intestinal tract, brain, nervous system, skin, bone and thyroid^14,15^. Also, nitrite ion is a major hazardous pollutant in wastewater production from nuclear power plants^16^. Therefore, it is highly desirable to develop a selective and sensitive method for detection and monitoring toxic HQ, CC, and NT to protect the public health and the environment^17^. Furthermore, there is always a challenge in simultaneously sensing of HQ, CC, and NT at traditional electrodes due to overlapping of their oxidation peaks. So, it is important to elaborately design a modified surface to be capable of avoiding fouling from the resulting oxidative products. To address these obstacles, numerous studies were introduced to explore new materials for sensitive and selective detection of HQ, CC, and NT^18,19^. Electrochemical methods among several methods have obtained noticeable interest due to their advantages such as simple preparation, low cost, fast response, sensitive and good analytical performance^20,21^. Different methods are applied for determination of HQ, CC and NT including spectrophotometric, high-performance liquid chromatography (HPLC), gas chromatography and so on^22–24^.

Poly(3,4-ethylenedioxythiophene) (PEDOT) is a conducting polymer and is used extensively in modification of electrode surfaces for sensing applications^25,26^. PEDOT modified electrodes are characterized by robust films, good electrocatalytic activity, high rate of electron transfer when used as electrode material, and excellent stability. Further, PEDOT possesses excellent environmental stability, biocompatibility, low band gap, excellent electrochemical activity, and high electric conductivity^27,28^. The thiophene ring in PEDOT has high electron affinity and aromaticity. PEDOT modified electrodes are used for detection of different compounds such as phenolic compounds, dopamine, uric acid, and morphine^29–31^.

Graphene oxide (GRO) is used for the fabrication of modified electrodes for sensing applications^32,33^. GRO morphology is characterized by a large surface area and plenty of functional groups (oxygen-containing groups) on its surface. Carbon sheets of GRO are protected from restacking and agglomeration by the presence of oxygen-containing groups on their surfaces^34^. These functional groups help GRO to disperse in solvents and water^35,36^. Also, they act as combining sites for graphene oxide-based composites. Combination of polymer with GRO is advantageous due to poor conductivity of GRO^37^.

Multiwalled carbon nanotubes (CNTs) are nanostructured materials widely used for electrode surfaces modification. CNTs are characterized by special rearrangement in the σ–π hybridization and large specific surface area^38^. Besides, they have good electrical conductivity, excellent electron transfer rate, high electrocatalytic activity, anti-fouling ability, high thermal and chemical stability^39^. CNTs have hexagonal structures along their surfaces but at the tube ends they have pentagon structures which are responsible about their reactivity^40^. CNTs are employed successfully in electrochemical sensing applications^33,41^. Further, the presence of CNTs between the GRO sheets can act as a conducting electrical network; this significantly facilitates the oxidation of target species. Also, the conductivity of the carbon nanostructures can be improved by forming composites with conducting polymers.

The outstanding synergism between the modifiers; PEDOT, CNTs, and GRO with their distinctive characteristics, enhances the electrochemical oxidation current responses of HQ, CC, and NT. Each of the sensor constituents shares in the enhancement of its performance. PEDOT film is a conductive firm film with excellent stability, good electrocatalytic activity and high electron transfer rate. GRO has a large surface area and plenty of functional groups (oxygen-containing groups) on its surface which act as reactive sites for binding and nucleation of target species at its surface. CNTs offer large effective surface area, high edge plane/basal plane ratio, high sorption capacity, penetration ability, surface adsorption and excellent electrical conductivity. Further, the incorporated CNTs can serve as an electrical conducting network between the GRO sheets, they greatly facilitate the electrochemical oxidation of HQ, CC, and NT.

For best of our knowledge only two papers cited the determination of HQ, CC, and NT simultaneously in their mixture. Here, in the present work, we introduce a novel electrochemical sensor; GC/PEDOT/CNT-GRO for simultaneous detection of three environmental contaminants namely HQ, CC, and NT in real water samples. The sensor is fabricated by modifying the surface of glassy carbon electrode by two successive thin films from PEDOT and a mixture of CNT-GRO. This increases the composite surface area, the contact area with analytes and improves the electrocatalytic behavior of the composite toward oxidation of HQ, CC, and NT. Under optimized conditions, the sensor in wastewater sample shows excellent catalytic effect for simultaneous determination of HQ, CC, and NT in the concentration ranges (0.04 → 100 µM), (0.01 → 100 µM) and (0.05 → 120 µM) with low detection limits of 8.5 nM, 3.8 nM and 6.1 nM, respectively. The combined effect of large conductive surface area and excellent catalytic activity of PEDOT/CNT-GRO nanocomposite improves the sensor’s performance. The GC/PEDOT/CNT-GRO modified electrode offers good sensitivities and low detection limits for HQ, CC, NT compared to other modified electrodes cited in the literature. Good reproducibility, repeatability and perfect storage stability are the features of the sensor. The sensor exhibits excellent selectivity, and insignificant interference from common species present in wastewater samples. Acceptable recovery rate is obtained for practical application of the sensor in wastewater samples for HQ, CC, and NT determination.

## Experimental section

### Chemicals

CNTs (> 90% carbon basis, OD/ID × L: 10–15 nm/2–6 nm × 0.1–10 μm), graphite powder, 3,4-ethylenedioxy-thiophene (EDOT), tetra butyl ammonium hexafluoro phosphate (Bu_4_NPF_6_), acetonitrile, H_2_SO_4_, KMnO_4_, DMF, hydroquinone (HQ), catechol (CC), KNO_2_ (NT), KH_2_PO_4_, K_2_HPO_4_, H_3_PO_4_, and KOH are purchased from Sigma-Aldrich Chem. Co. (Milwaukee, WI. USA). Supplement Table 1 summarizes the instruments used in this work.

### Preparation of GRO

Hummer’s method is used to prepare GRO. Briefly, to prepare the pre-oxidized graphite (98%), H_2_SO_4_ (40 mL), K_2_S_2_O_8_ (8.4 g) and P_2_O_5_ (8.4 g) are used to cure 10 g of highly pure graphite. Then in an ice bath 3 g of dried pre-oxidized graphite is stirred with 115 mL of concentrated H_2_SO_4_ for 10 min. This is followed by gradual addition of 15 g of KMnO_4_ while stirring for a period of two hours. After dilution with water and treatment with H_2_O_2_, a bright yellow color suspension is obtained and filtered. Lastly, 1:10 (v/v) HCl-solution is used to wash the suspension, and is dried in an oven overnight at 80 °C^42^.

### Preparation of the sensor

The GC/PEDOT/CNT-GRO sensor is prepared as follows: (GC/PEDOT) is prepared from a solution of 0.001 M EDOT and 0.05 M Bu_4_NPF_6_ in acetonitrile by electrochemical polymerization^43,44^. This step is accomplished by using cycling voltammetry (CV) in the potential range (− 1.5 V to 2 V) for 3 cycles at scan rate of 100 mV s^−1^^45^. Then 10 μL from a suspension mixture of (0.5 mg GRO-0.5 mg CNTs/1.0 mL DMF) is drop-cast over the previous modified electrode surface GC/PEDOT. Then the electrode is dried in an oven at 50 °C for 10 min.

We run CV experiments in 1.0 mM K_3_[Fe(CN)_6_] system using the modified electrodes to calculate their electrochemical effective surface areas as mentioned elsewhere^46^. The values of the active surface areas for bare GC, GC/GRO, GC/PEDOT, GC/PEDOT/GRO, GC/CNT, GC/CNT-GRO, GC/PEDOT/CNT, and GC/PEDOT/CNT-GRO are: 0.0474 cm^2^, 0.064 cm^2^, 0.0902 cm^2^, 0.0961 cm^2^, 0.102 cm^2^, 0.129 cm^2^, 0.1335 cm^2^, and 0.1462 cm^2^, respectively, Supplement Fig. 1A–H. The active surface areas of the modified electrodes increased in the following order GC < GC/GRO < GC/PEDOT < GC/PEDOT/GRO < GC/CNT < GC/CNT-GRO < GC/PEDOT/CNT < GC/PEDOT/CNT-GRO. Modification of GC electrode surface increases the composite surface area, the contact area with analytes and improves the electrocatalytic behavior of the composite toward oxidation of HQ, CC, and NT. Thus, the oxidation current response of the analyte under investigation increases with increasing the electroactive surface area.

### Preparation of real sample

We obtained water samples from river, the sample is pretreated as mentioned elsewhere^47^. The wastewater sample is diluted by 0.1 M PBS/pH 7.0 one time; 10.0 mL of diluted sample is transferred to the electrolytic cell. To reach the desired concentrations, the prepared solution 0.1 mM for each of HQ, CC, and NT mixture/0.1 M PBS/pH 7.0 is spiked with pre-calculated volumes in diluted wastewater sample. Then, the experiments are studied using DPV technique.

### SEM and Raman analyses

Scanning electron microscopy (SEM) is used to investigate the surface structural/morphological images of the studied composites. We prepared thin films from GC/PEDOT, GC/PEDOT/GRO and GC/PEDOT/CNT-GRO over GC sheets as mentioned earlier, the films were introduced directly to the measurements without further treatment.

Surface enhanced Raman spectroscopy (SERS) is used to obtain the vibrational modes of the functional groups of the composite film. We prepared the composite sample GC/PEDOT/CNT-GRO over a GC sheet as mentioned earlier, and then the experiment is conducted on the prepared surface to record the Raman spectrum.

## Results and discussion

### Surface characterization of the modified electrodes

**Figure 1:**
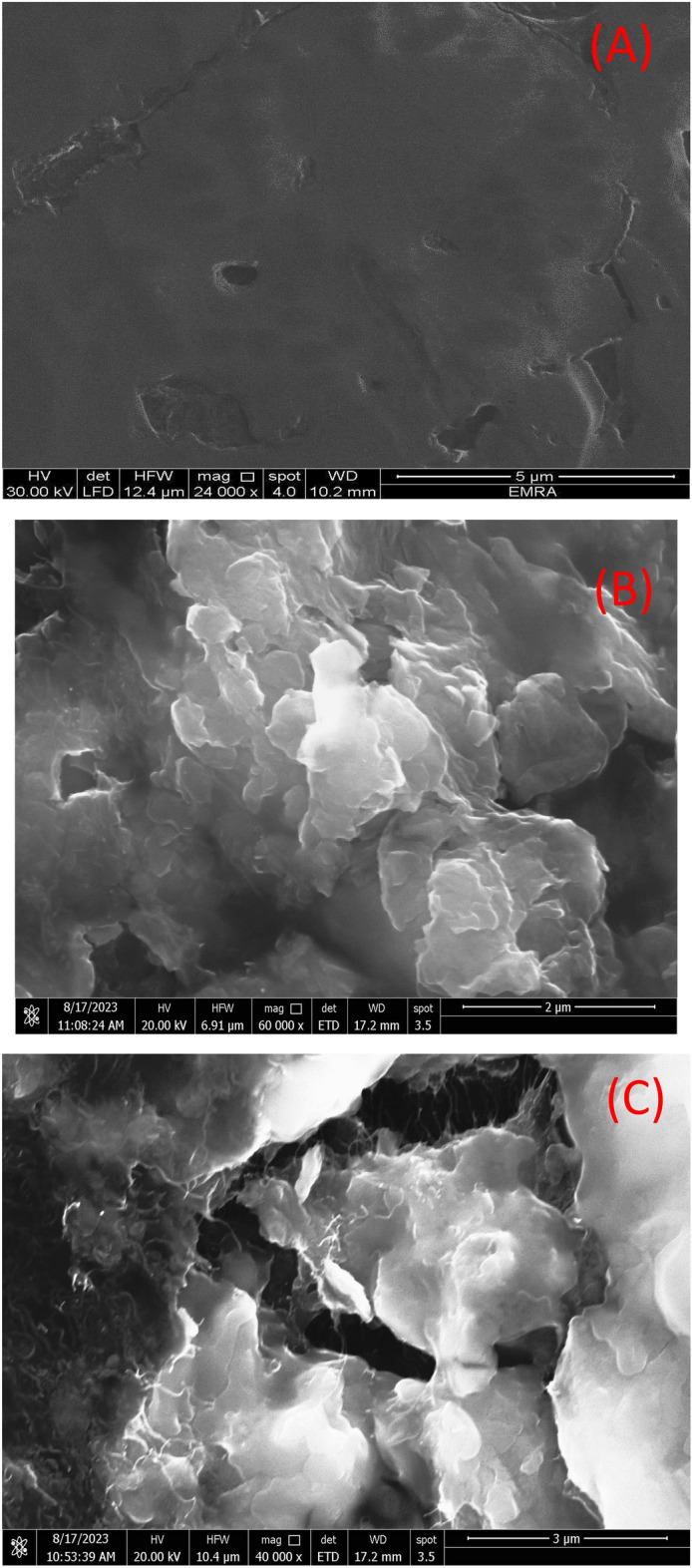

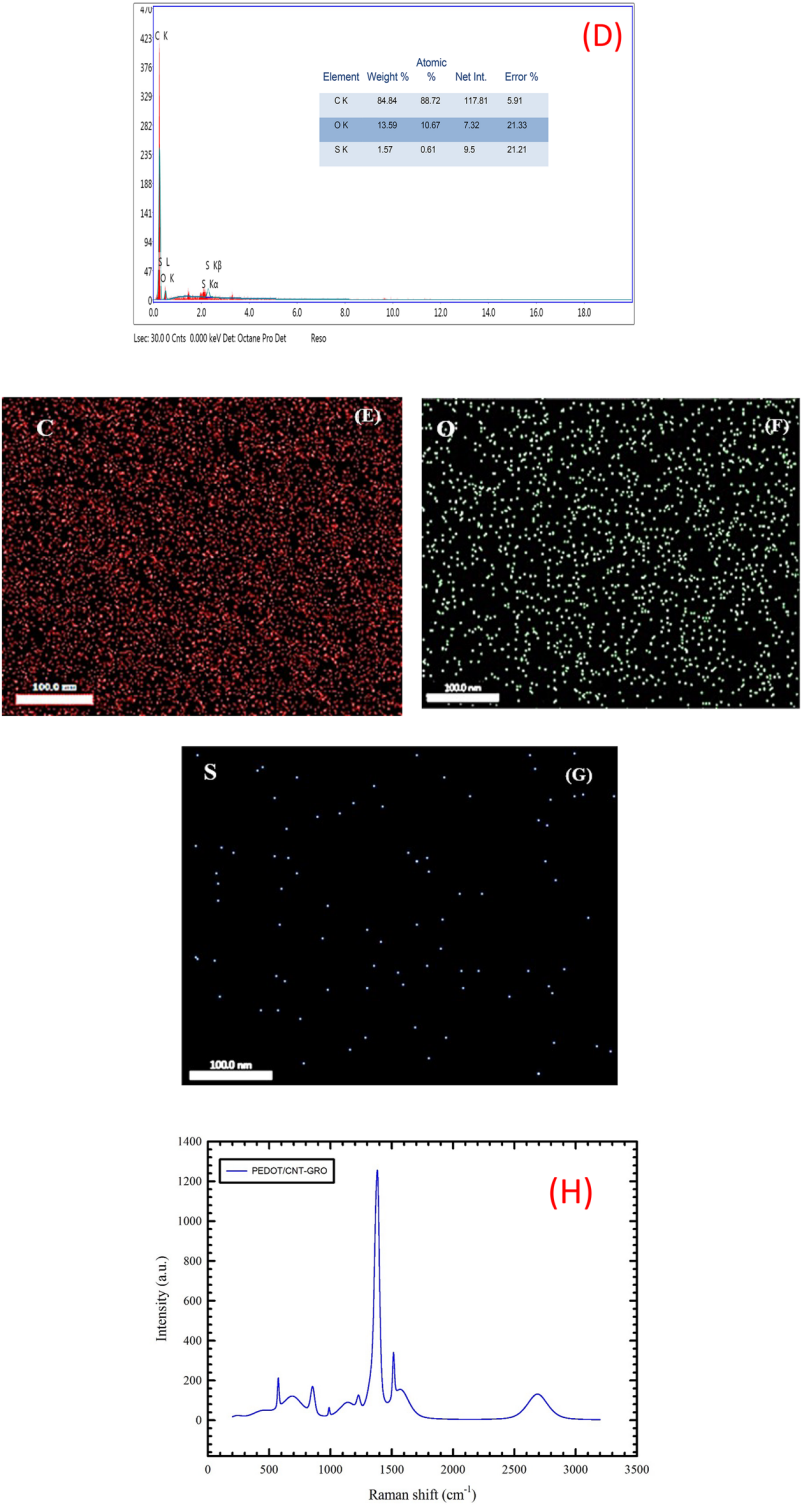
(**A**–**C**) SEM image of GC/PEDOT, GC/PEDOT/GRO and GC/PEDOT/CNT-GRO, (**D**) EDX of GC/PEDOT/CNT-GRO, (**E**–**G**) Elemental mapping of PEDOT/CNT-GRO composite, and (**H**) Raman spectroscopy for PEDOT/CNT-GRO.

The surface morphology of GC/PEDOT, GC/PEDOT/GRO and GC/PEDOT/CNT-GRO are examined by scanning electron microscopy. The morphology of PEDOT modified glassy carbon electrode surface shows a dense compact and homogeneous structure^48,49^, Fig. 1A. GRO sheets are characterized by thick flat flake layers, irregular shapes and rough surfaces as shown in Fig. 1B. The unwrinkled and disordered structures of GRO sheets are due to the presence of functional groups containing oxygen atoms over their surfaces. Figure 1C shows the SEM of GC/PEDOT/CNT-GRO, where the CNTs are thoroughly mixed with the graphene oxide sheets. Due to noncovalent π–π stacking interactions, the GRO sheets are surrounded by the CNTs tubular networks^50^. The presence of all elements in the composite are identified from EDX and mapping of the GC/PEDOT/CNT-GRO as shown in Fig. 1D–G.

The FTIR technique is used to analyze the structural components of PEDOT, CNT-GRO, and PEDOT/CNT-GRO. Supplement Fig. 2A shows the FTIR spectrum of PEDOT bands. Characteristic peaks are observed below 1600 cm^−1^. The peaks at 1584 cm^−1^ and 1366 cm^−1^ are assigned to C=C and C–C stretching vibrations, while the bands at 1183 cm^−1^, 1135 cm^−1^, 1100 cm^−1^, and 1056 cm^−1^ are attributed to the C–O–C bending vibrations in the ethylenedioxy group. The bands at 933 ~ 678 cm^−1^ are characteristic for stretching vibrations of the C-S-C bond in the thiophene ring, indicating a successful formation of PEDOT in the polymerization reaction^51,52^. Supplement Fig. 2B shows the FTIR spectrum of CNT-GRO composite. The spectrum consists of vibrational groups of the GRO that includes carbonyl (C=O), aromatic (C=C), carboxyl (COOH), epoxy (C–O–C), and hydroxyl (O–H) groups. A sharp peak appeared at 3420 cm^−1^ corresponds to the hydroxyl group (O–H)^53^. The peaks at 1697 cm^−1^ and at 1300 cm^−1^ are attributed to the ketone group (C=O) and to C–O–C ^53^ respectively. Additionally, the peak at 1577 cm^−1^ is attributed to the characteristic backbone C=C skeletal stretching of CNTs^54^. Supplement Fig. 2C shows the FTIR spectrum of PEDOT/CNT-GRO. The characteristic vibrational peaks that appeared are for PEDOT, CNTs, and GRO such as C–S–C at 887 ~ 684 cm^−1^, C–C at 1381 cm^−1^, C–O–C at 1184–1048 cm^−1^, C=C at 1579 cm^−1^, OH at 3419 cm^−1^, and C=O at 1629 cm^-1^, these peaks are slightly shifted compared to PEDOT and CNT-GRO spectra due to interactions between the composite components.

The Raman fingerprints of PEDOT, CNTs and GRO have been previously investigated^55–57^. The vibrational modes of PEDOT are located at 1514 cm^−1^, 1390 cm^−1^, and 1272 cm^−1^, and assigned to the Cα = Cβ asymmetrical, Cα = Cβ symmetrical stretching, and Cα – Cα inter-ring stretching vibrations, respectively, Supplement 3A. The Raman spectrum of the CNTs shows the D-band peak between 1300 cm^−1^ and 1400 cm^−1^ spectral range, also known as the disorder band, is due to scattering from sp2 carbon containing defects precisely appeared at 1359 cm^−1^. The peak between 1500 cm^−1^ and 1600 cm^−1^ region called the G-band appeared at 1573 cm^−1^. A characteristic peak at 2688 cm^−1^ is also obtained and commonly written as D′ or 2D band, which depends upon the strain or stress applied to the carbon nanotube, Supplement 3B. The Raman spectrum of GRO displayed two unambiguous bands at 1348 cm^−1^ and 1585 cm^−1^ that are due to the D and G peaks, these bands arose from the lattice defects in the atomic crystal structure of the carbon material, Supplement 3C. To investigate the carbon structure and to confirm the presence of all the individual components in the composite, Raman spectrum of PEDOT/CNT-GRO is recorded (Fig. 1H). The characteristic peaks of the PEDOT are observed at 1381 cm^−1^ and 1514 cm^−1^ for the asymmetric and symmetric C=C stretching vibrations^58^. A characteristic peak of CNTs at 2688 cm^−1^ is obtained and commonly is written as D’ or 2D band, which depends upon the strain or stress applied to the carbon nanotubes^59,60^. The GRO nanosheets exhibit the characteristic peaks at 1381 cm^−1^ (D band) for in-plane bond stretching of sp^2^ carbon atoms and at 1568 cm^−1^ (G band) for defects of structure and lattice distortion^61^. Supplement Fig. 4A displays the XRD pattern of GRO prepared by Hummer's method, where the GRO shows a distinctive diffraction peak at 10° corresponding to the (001) plane.

Supplement Fig. 4B displays the XRD pattern of PEDOT/CNT-GRO where a major diffraction peak appeared at 2θ = 26°, that is attributed to the (002) plane. The sharpness of this peak is indicative of the structure of multiwalled carbon nanotubes^62^. Also, there is another peak for CNTs which appeared at 42.7°, that is corresponding to (100) plane. PEDOT should show a relatively medium peak at around 2θ = 26°, which is overlapping in this case with the sharp peak of carbon nanotubes^63^. Besides, the pattern shows a diffraction peak appeared at 2θ = 10° for GRO which is assigned to the (001) plane^64^.

### Electrochemical impedance spectroscopy “EIS”

**Figure 2:**
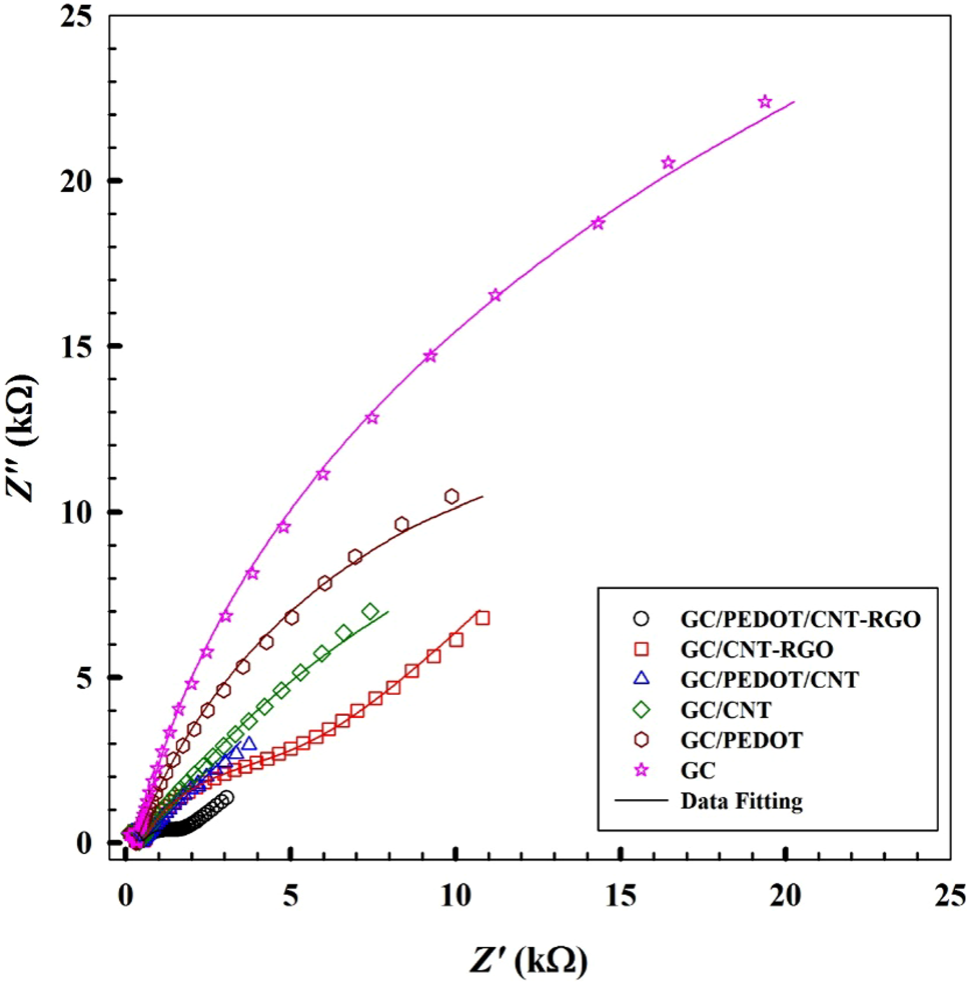
Typical impedance spectra presented in the form of the Nyquist plots for at bare GC, GC/PEDOT, GC/CNT, GC/CNT-GRO, GC/PEDOT/CNT, and GC/PEDOT/CNT-GRO, in 1.0 mM of K_3_[Fe(CN)_6_]/0.1 M PBS/pH 7.0. (Symbols and solid lines represent the experimental measurements and the computer fitting of impedance spectra, respectively).

Electrochemical impedance spectroscopy is an effective technique to investigate the interface properties of the modified electrodes. A suggested illustration of the surface modification and its corresponding variation in the charge transfer kinetics and capacitive components of the system can be clarified by the aid of EIS. EIS experiments are carried out in 1.0 mM K_3_[Fe(CN)_6_]/0.1 M PBS/pH 7.0 at applied potential of 0.1 V (vs. Ag/AgCl) in the frequency range of 0.1 Hz to 100 kHz at bare GC, GC/PEDOT, GC/CNT, GC/CNT-GRO, GC/PEDOT/CNT, and GC/PEDOT/CNT-GRO electrodes. Figure 2 shows the impedance spectra in the form of Nyquist plots for the different working electrodes. The software used for EIS data fitting is supplied with the instrument. Supplement Fig. 5 shows three equivalent circuits used for fitting the EIS experimental data. The first equivalent circuit is used for fitting the GC electrode data, the second equivalent circuit is used for fitting the GC/PEDOT and GC/CNT electrodes data, and the third equivalent circuit is used for fitting the GC/PEDOT/CNT, GC/CNT-GRO and GC/PEDOT/CNT-GRO electrodes data. These circuits contain various elements: *R*_s_ and *R*_ct_ are the solution resistance, and the charge transfer resistance, respectively;* R*_2_ and *R*_3_ represent the different film layers resistances_._ Capacitance is represented by C. Also, two constant phase elements *Y*_1_° and *Y*_3_° are used to describe the capacitance, surface inhomogeneity and roughness of surface; n, m are their corresponding exponents (with values < one). Charge diffusion from bulk of solution to electrode surface is represented by Warburg impedance *Y*_2_° **(**W). A quasi-semicircle part of the plot with large diameter at higher frequency region and a linear part at lower frequency region are depicted in Fig. 2 at bare GC corresponding to electron transfer-controlled and diffusion-controlled processes, respectively. The charge transfer resistance decreased for the working electrodes in the following order: bare GC > GC/PEDOT > GC/CNT > GC/CNT-GRO > GC/PEDOT/CNT > GC/PEDOT/CNT-GRO, manifesting relatively lower charge transfer resistance and fast charge transfer kinetics upon modification of GC electrode. GC has a higher charge transfer resistance 53.72 kΩ. Also, there is a significant decrease in the total impedance in the Nyquist plot of GC/PEDOT/CNT-GRO compared to bare GC depicting the effective performance and catalytic activity of the proposed sensor. Table 1 illustrates a summary of the EIS fitting data for the previously mentioned electrodes. Comparing the modified electrodes GC/CNT-GRO, GC/PEDOT/CNT and GC/PEDOT/CNT-GRO. A noticeable decrease in the value of *R*ct and *R*_2_ are observed upon modification of the GC with PEDOT/CNT-GRO, confirming enhanced electron transfer process. Also, an increase in the value of Warburg W is observed upon modification reflecting fast diffusion kinetics. The components of the nanocomposite offer enhanced conductivity, and improved surface area of the proposed surface. An increase in the ionic accumulation at the surface of the proposed surface is confirmed by the high value of constant phase element* Y*_3_° at GC/GC/PEDOT/CNT-GRO.

**Table 1:** EIS fitting data corresponding to results in Fig. 2

	*R*_s_ (Ω)	*R*_ct_ (kΩ)	*Y*_1_° (n) (Q1) × 10^–5^ (μMho s^n^)	*Y*_2_° (W) × 10^–4^ (μMho s^½^)	*R*_2_ (Ω)	*C* × 10^–5^ (F)	*R*_3_ (kΩ)	*Y*_3_° (m) (Q2) × 10^–5^ (μMho s^n^)	χ^2^
GC	282	53.72	–	3.45	–	2.52	–	–	0.329
GC/PEDOT	237	13.3	7.08 (0.780)	–	458	–	–	9.30 0.926	0.0374
GC/CNT	373	12.9	10.5 0.698	–	514	–	–	1.34 0.978	0.0352
GC/CNT-GRO	789	13.0	1.43 0.750	1.28	366	1.19	1.01	4.25 0.891	0.0178
GC/PEDOT/CNT	369	8.87	9.46 0.784	2.91	318	1.30	0.734	3.99 0.932	0.0820
GC/PEDOT/CNT-GRO	687	0.455	1.64 0.760	7.13	269	1.23	0.964	9.62 0.930	0.0303

GC electrode data’ fitted with equivalent circuit 1.

GC/PEDOT and GC/CNT electrodes data’ fitted with equivalent circuit 2.

GC/PEDOT/CNT, GC/CNT-GRO and GC/PEDOT/CNT-GRO electrodes data’ fitted with equivalent circuit 3.

### Electrochemistry of the working electrodes

**Figure 3:**
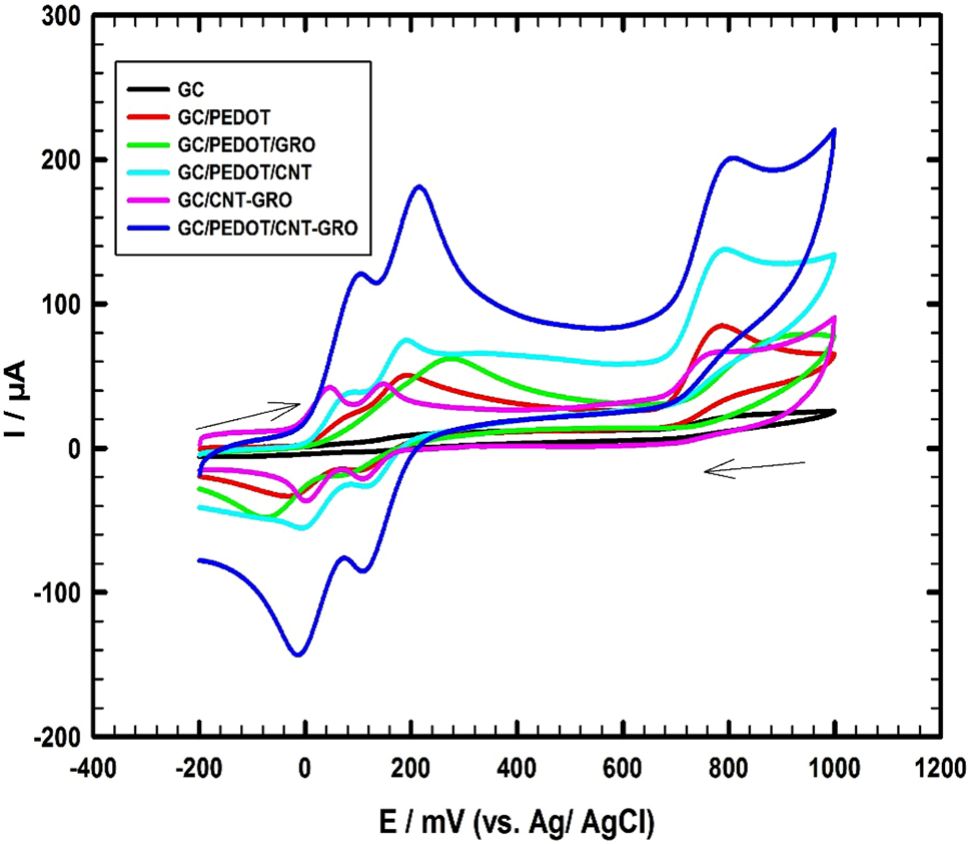
CVs of ternary mixture of 200 µM HQ, 150 µM CC, and 100 µM NT/0.1 M PBS (pH 7.0), scan rate 50 mV/s using different working electrodes.

The electrochemical behavior of HQ, CC, and NT/0.1 M PBS (pH 7.0) at modified GC electrodes, upon layer-by-layer modification of GCE surface, is evaluated using cyclic voltammetry mode (CV). Figure 3 displays the CVs of a ternary mixture containing 200 µM HQ, 150 µM CC, and 100 µM NT prepared in 0.1 M PBS recorded at the following working electrodes: bare GC, GC/PEDOT, GC/PEDOT/GRO, GC/CNT-GRO, GC/PEDOT/CNT and GC/PEDOT/CNT-GRO, with a scan rate of 50 mV/s. Supplement Fig. 6 shows the blanks for all the modified working electrodes. The electrochemical data for the oxidation of the studied analytes are summarized in Supplement Table 2.

**Figure 4:**
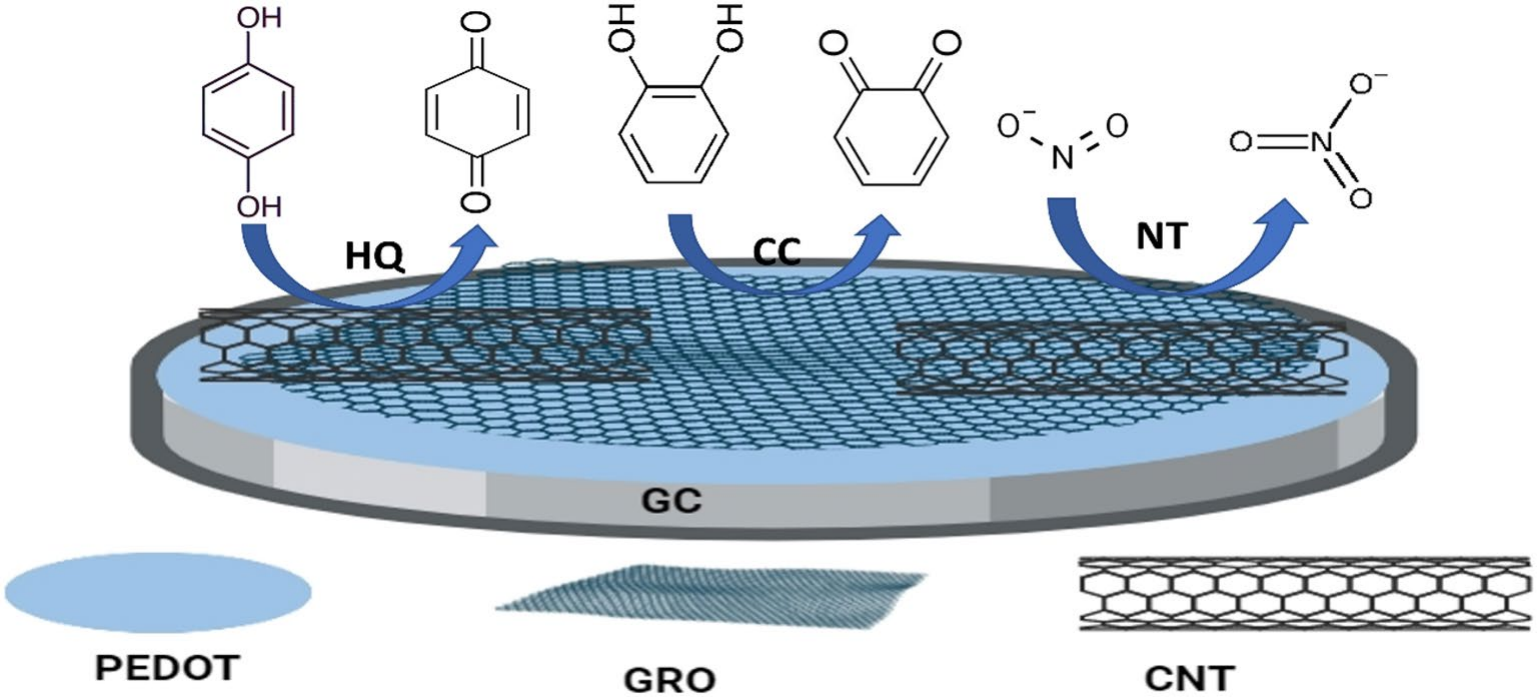
Schematic representation of GC/PEDOT/CNT-GRO modified electrode used for the electrochemical oxidation of HQ, CC, and NT.

Using the bare GCE or GC/PEDOT/GRO electrode, overlapped oxidation current signals for HQ and CC with low current response of NT are obtained for HQ, CC, and NT. Modifying the GC electrode surface with a thin film of PEDOT enhances the current signals and improves the resolution of the oxidation peaks compared to bare GC electrode. Then, modification of the GC electrode surface by two consecutive thin layers from PEDOT and CNTs, higher current responses are obtained for all the three analytes. The oxidation current responses are 40 μA, 31 μA, and 76 μA for HQ, CC, and NT at 83 mV 195 mV, and 791 mV, respectively. This improvement in current responses can be attributed to the synergistic effect between PEDOT and CNTs. The combination of these materials enhances the catalytic effect toward HQ, CC, and NT oxidation due to their good electric conductivity, and large surface area. Modification of the GC electrode surface by one layer of CNT-GRO mix, the oxidation current responses are 27 μA, 28 μA, and 32 μA for HQ, CC, and NT at 41 mV 145 mV, and 745 mV, respectively. But further modification of the GC electrode surface with two thin layers of PEDOT and CNT-GRO mixture enhances the sensor sensitivity and increases the oxidation current responses up to 106 μA, 82 μA, and 111 μA at 96 mV, 213 mV and 798 mV, respectively. An increase in the anodic current responses for HQ, CC, and NT is obtained with 5.1, 4.1, and 2.3 times higher compared to GC/PEDOT electrode, respectively. This is due to high electric conductivity of PEDOT and CNTs with a large active surface area of the PEDOT/CNT-GRO composite which resulted in acceleration of the electron transfer rate between the studied analytes and the composite surface. Thus, the synergistic effect between the modifiers GRO, CNTs, and PEDOT increases the electro-catalytic activity of the PEDOT/CNT-GRO composite toward the oxidation of HQ, CC, and NT. Besides, the conducting structure of the nanocomposite improves the voltametric separation between the HQ, and CC isomers that oxidize at nearly similar potentials under diffusion conditions and increases their oxidation current responses. The oxidation mechanisms of HQ, CC, and NT/0.1 M PBS (pH 7.0) using the GC/PEDOT/CNT-GRO electrode are illustrated in Fig. 4

### Effect of scan rate

The effect of varying the scan rate (10 to 100 mV s^−1^) on the current responses of 100 µM HQ, 100 µM CC, and 120 µM NT/0.1 M PBS (pH 7.0) using a GC/PEDOT/GRO-CNT electrode is investigated via CV mode, Supplement Fig. 7. The inset A shows linear relationships between I_p_ and ν^1/2^ for HQ, CC, and NT, indicating diffusion-controlled processes. The corresponding equations for the linear relations of HQ, CC, and NT can be summarized as follows:

$$\begin{aligned} {\text{I}}_{{\text{p}}} (\upmu {\text{A}}) & \, = - {1}0.{64 } + {6}.0{5}\nu^{1/2} \left( {{\text{mVs}}^{{ - {1}}} } \right)^{1/2} \;\;\;\; \left( {{\text{R}}^{{2}} = \, 0.{997}} \right)\;{\text{ for }}\;{\text{HQ}} \\ {\text{I}}_{{\text{p}}} (\upmu {\text{A}}) \, & = - {14}.{4 } + {7}.{71}\nu^{1/2} \left( {{\text{mVs}}^{{ - {1}}} } \right)^{1/2} \;\;\;\;\left( {{\text{R}}^{{2}} = \, 0.{99}0} \right) \, \;{\text{for }}\;{\text{CC}} \\ {\text{I}}_{{\text{p}}} (\upmu {\text{A}}) \, & = \, - {9}.{13 } + {8}.0{4}\nu^{1/2} \left( {{\text{mVs}}^{{ - {1}}} } \right)^{1/2} \;\;\;\; \left( {{\text{R}}^{{2}} = \, 0.{991}} \right)\;{\text{ for }}\;{\text{NT}} \\ \end{aligned}$$

Also, linear relationships between log I_p_ and log *v* for HQ, CC, and NT are obtained, verifying that the processes are diffusion controlled, Inset B of Supplement Fig. 7.

$$\begin{aligned} {\text{Log I}}_{{\text{p}}} (\upmu {\text{A}}) & \, = \, 0.{33 } + 0.{\text{67 log}}\nu \left( {{\text{Vs}}^{{ - {1}}} } \right), \;\;\;\left( {{\text{R}}^{{2}} = \, 0.{994}} \right)\;{\text{ for }}\;{\text{HQ}} \\ {\text{Log I}}_{{\text{p}}} (\upmu {\text{A}}) & \, = \, 0.{34 } + 0.{\text{71 log}}\nu \left( {{\text{Vs}}^{{ - {1}}} } \right), \;\;\;\left( {{\text{R}}^{{2}} = \, 0.{991}} \right) \, \;{\text{for }}\;{\text{CC}} \\ {\text{Log I}}_{{\text{p}}} (\upmu {\text{A}}) & \, = \, 0.{64 } + 0.{\text{61 log}}\nu \left( {{\text{Vs}}^{{ - {1}}} } \right),\;\;\; \left( {{\text{R}}^{{2}} = \, 0.{992}} \right)\;{\text{ for }}\;{\text{NT}} \\ \end{aligned}$$

The correlation slopes are 0.67, 0.71, 0.61 for HQ, CC, and NT respectively, close to the theoretical value of 0.5, indicating that the oxidation reactions are diffusion controlled processes^65^.

For the reversible electrochemical process, according to Laviron theory^66^, a graph of E_p_ = f (log *v*) shows two linear lines with slopes equal to − 2.3RT/*v*nF for the cathodic peak and 2.3RT/(1 − *v*)nF for the anodic peak. The charge transfer coefficient (α) can be calculated based on the slopes of the two linear lines of E_p_ versus log *v* relation using the following equation:

$$\frac{{k}_{a}}{{k}_{c}}=\frac{\mathrm{\alpha }}{1-\mathrm{\alpha }}$$

where *k*_a_ and *k*_c_ are the slopes of the linear lines for E_pa_ versus log *v*, and E_pc_ versus log *v*, respectively.

The relations between the anodic peak potential E_pa_ and the cathodic peak potential E_pc_ for HQ and CC versus the logarithm of the scan rate are depicted in inset C of Supplement Fig. 7 and can be represented by the following linear relationships:

$$\begin{aligned} {\text{E}}_{{{\text{pa}}}} \left( {\text{V}} \right) & \, = \, 0.{148 } + \, 0.0{\text{45 Log}}\nu \left( {{\text{Vs}}^{{ - {1}}} } \right) \;\;\;\; \left( {{\text{R}}^{{2}} = \, 0.{984}} \right)\;{\text{ for }}\;{\text{HQ}} \\ {\text{E}}_{{{\text{pc}}}} \left( {\text{V}} \right) & \, = \, - 0.0{31 }{-} \, 0.0{\text{36 Log}}\nu \left( {{\text{Vs}}^{{ - {1}}} } \right)\;\;\;\; \left( {{\text{R}}^{{2}} = \, 0.{994}} \right) \, \;{\text{for}}\;{\text{ HQ}} \\ {\text{E}}_{{{\text{pa}}}} \left( {\text{V}} \right) & \, = \, 0.{232 } + \, 0.0{\text{41 Log}}\nu \left( {{\text{Vs}}^{{ - {1}}} } \right) \;\;\;\; \left( {{\text{R}}^{{2}} = \, 0.{986}} \right) \, \;{\text{for}}\;{\text{ CC}} \\ {\text{E}}_{{{\text{pc}}}} \left( {\text{V}} \right) & \, = \, 0.0{98}{-} \, 0.0{\text{22 Log}}\nu \left( {{\text{Vs}}^{{ - {1}}} } \right) \;\;\;\; \left( {{\text{R}}^{{2}} = \, 0.{987}} \right) \, \;{\text{for}}\;{\text{ CC}} \\ \end{aligned}$$

Thus, α values were calculated and found to be 0.55 and 0.65 for HQ and CC, respectively. Using the slope of E_p_ versus log *v*, the calculated n values are equal to 2.21 and 2.36 for HQ and CC, respectively, indicating that two electrons are involved in the electrochemical oxidation of HQ and CC.

Also, a linear relationship between the anodic peak potential (E_pa_) and the natural logarithm of the scan rate (log *v*) for NT is depicted in inset D of supplement Fig. 7 and is represented by the following linear regression equation.

$${\text{E}}_{{{\text{pa}}}} \left( {\text{V}} \right) \, = \, 0.{831}{-} \, 0.0{\text{38 Log}} \nu \left( {{\text{Vs}}^{{ - {1}}} } \right) \;\;\;\; \left( {{\text{R}}^{{2}} = \, 0.{987}} \right)\;{\text{ for}}\;{\text{ NT}}$$

For a totally irreversible electrode process, the relationship between E_pa_ versus log *v* is expressed as follows by Laviron^67^:

$${\text{E}}_{{\text{p}}} \left( {\text{V}} \right) \, = \, \left( {{2}.{\text{3RT}}/\alpha {\text{nF}}} \right){\text{ log}}\nu \left( {{\text{V}}/{\text{s}}} \right) \, + {\text{Constants}}$$

where R = 8.314 J/K mol, F = 96,480 C/mol, T = 298 K, and n is the total number of electrons exchanged. α is the electron transfer coefficient. α can be derived using the following formula^68^.

$$\alpha \, = { 47}.{7 }/ \, \left( {{\text{E}}_{{\text{P}}} - {\text{E}}_{{{\text{P}}/{2}}} } \right)$$

where E_P/2_ is the potential where the current is at half the peak value. α was calculated to be 0.793, The value of n can be determined as 1.95 = 2 using the slope of E_p_ and log *v*.

### Effect of pH

**Figure 5:**
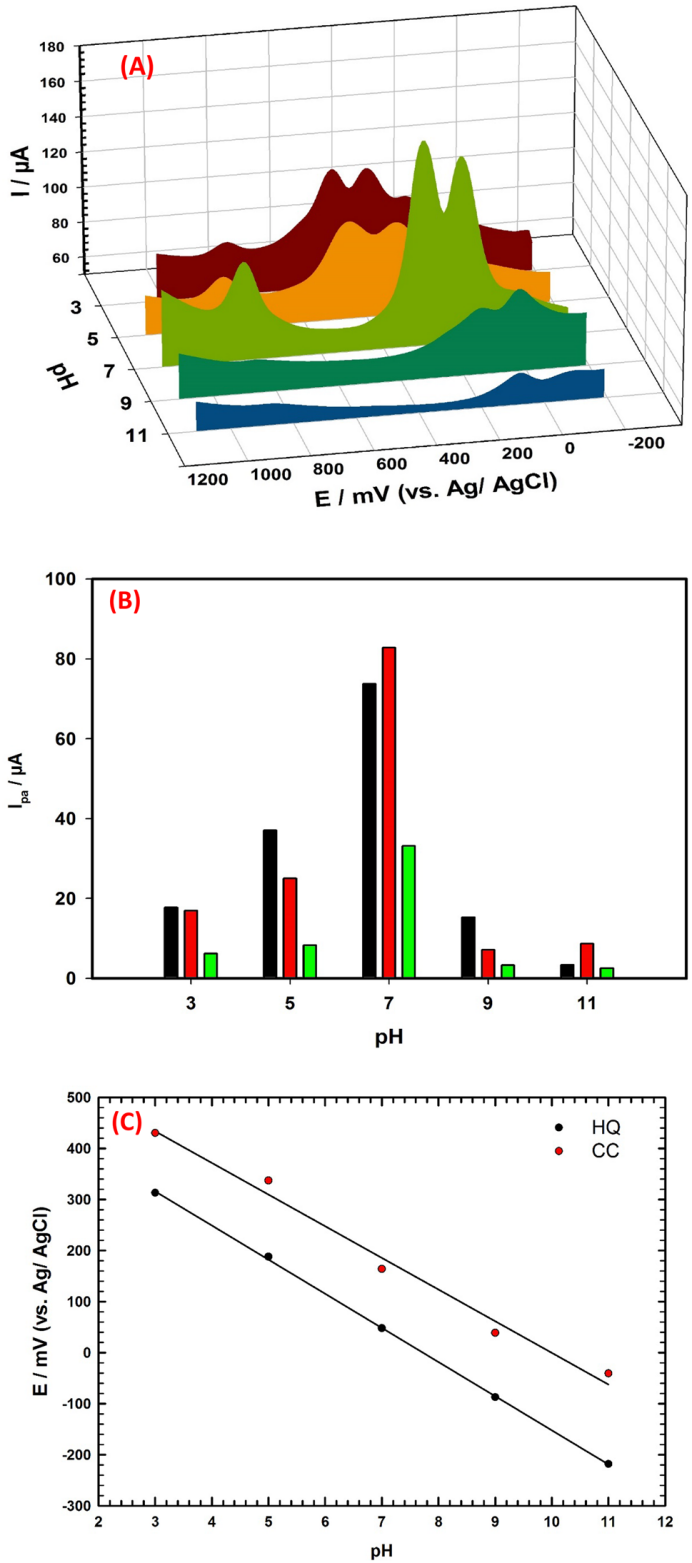
(**A**) DPVs of ternary mixture of 200 µM HQ, 150 µM CC, and 100 µM NT/0.1 M PBS with different pH values, scan rate 20 mV/s using GC/PEDOT/CNT-GRO electrode. (**B**) Relations between oxidation peak currents and pH for CC, HQ, and NT. (**C**) Relations between oxidation peak potentials and pH for CC and HQ.

The performance of the electrochemical sensor is significantly affected by the pH of the electrolyte. To investigate the effect of pH on the simultaneous detection of HQ, CC, and NT in their mixture, DPV measurements are performed. The DPV responses of 200 µM HQ, 150 µM CC, and 100 µM NT are recorded in 0.1 M PBS within the pH range of 3 to 11, as shown in Fig. 5A. The results reveal that the oxidation peak currents of HQ, CC, and NT increase with increasing pH, and they reach their maximum value at pH 7.0 as shown in Fig. 5B. Therefore, pH 7.0 is chosen as the optimal condition for the subsequent experiments. In strongly acidic medium the nitrite is not stable, where it is easily undergoing a disproportionation reaction which leads to a decrease of nitrite oxidation current response^69^. The presence of hydrogen ions is necessary for the oxidation of nitrite^70^. Therefore, when the pH is higher than 7.0, the number of hydrogen ions decreased in basic medium, thus the nitrite oxidation is inhibited, and its oxidation peak current decreased, Fig. 5B. Several studies mentioned proposed mechanisms for nitrite oxidation. The mechanism suggests an electrochemical step (1) with the formation of NO_2_ and followed by a chemical disproportionation reaction (2) and formation of NO_3_^−^ (3) as follows^71^:

1 $${\text{2NO}}_{{2}}^{ - } \rightleftarrows {\text{2NO}}_{{2}} + {\text{ 2e}}^{ - }$$

2 $${\text{2NO}}_{{2}} + {\text{ H}}_{{2}} {\text{O}} \to {\text{2H}}^{ + } + {\text{ NO}}_{{2}}^{ - } + {\text{ NO}}_{{3}}^{ - }$$

3 $${\text{NO}}_{{2}}^{ - } + {\text{ H}}_{{2}} {\text{O}} \to {\text{NO}}_{{3}}^{ - } + {\text{ 2H}}^{ + } + {\text{ 2e}}^{ - }$$

The HQ, and CC compounds pKa values are 9.85, and 9.4, respectively^72^. Thus, at pH 7.0 there are good interactions between the partially negative charges oxygen atoms over the GRO surface (functional groups containing oxygen) and the protonated forms of HQ and CC compounds. At pH value lower or higher than 7.0, there are weak interactions between the oxygen atoms over GRO surface and the HQ and CC compounds. This is due to the presence of protons in acidic medium competing with the protonated compounds, and in basic medium the protonated compounds became neutral i.e., deprotonation of the protonated compounds occurred with the existence of OH^-^ ions in the medium^18^.

Figure 5C illustrates that the anodic peak potentials (E_p_) for HQ and CC shift negatively with increasing pH from 3 to 11, indicating the participation of protons in the electrochemical redox processes^73,74^. Additionally, the E_p_ exhibits linear relationships with increasing pH for HQ and CC and can be represented by the following equations:

$$\begin{aligned} {\text{E}}_{{{\text{p}}\;{\text{ HQ}}}} \left( {{\text{mV}}} \right) & \, = { 478 }{-}{ 62}.{\text{6 pH}}\;\;\;\;{\text{R}}^{{2}} = \, 0.{997} \\ {\text{E}}_{{{\text{p}}\;{\text{CC}}}} \left( {{\text{mV}}} \right) & \, = { 611 }{-}{ 6}0.{\text{9 pH}}\;\;\;\; {\text{R}}^{{2}} = \, 0.{987} \\ \end{aligned}$$

The slopes of the regression equations are close to the Nernst theoretical value of 59 mV/pH, suggesting that the electrochemical redox reaction for each of HQ and CC at GC/PEDOT/CNT-GRO is two-protons and two-electrons process^75^. Thus, the pH study provides a valuable insight into the electrochemical behaviors of HQ, CC, and NT, it can aid in the development of more accurate and reliable electrochemical sensors for their detections.

### Reproducibility and stability

Reproducibility and stability are important parameters for precise and accurate analytical measurement. The relative standard deviation values (RSDs) of oxidation current responses for 100 µM HQ, 100 µM CC, and 120 µM NT in their mixture using three similar fabricated GC/PEDOT/CNT-GRO electrodes are 1.23%, 1.73%, and 0.89%, respectively. Further, the stability of the GC/PEDOT/CNT-GRO sensor in a mixed solution of 100 µM HQ, 100 µM CC, and 120 µM NT/0.1 M PBS is evaluated. The RSDs of oxidation current responses obtained after 25 continuous CV cycles for HQ, CC, and NT in their mixture are 2.08%, 1.29%, and 2.83%, respectively, Supplement Fig. 8A. Also, the stability of the GC/PEDOT/CNT-GRO sensor in low concentration mixed solution of 2 µM HQ, 5 µM CC, and 3 µM NT/0.1 M PBS is examined. The RSDs of oxidation current responses obtained after 15 continuous DPVs for HQ, CC, and NT in their mixture are 2.038%, 1.84%, and 1.28%, respectively, Supplement Fig. 8B.

Moreover, the GC/PEDOT/CNT-GRO electrode is stored for one month, the electrochemical current responses of HQ, CC, and NT maintain 95.3%, 98.2%, and 96.9% of their initial current values, respectively. These results suggest that the GC/PEDOT/CNT-GRO electrode has a good reproducibility, stability, and acceptable repeatability, making it a good choice for simultaneous detection of these pollutants species.

### Robustness

The robustness of this method is assessed by examining the impact of minor changes in the experimental conditions. Two parameters are studied, namely the time before running the experiment (2 min ± 20 s) and pH change (7.0 ± 0.2). The RSDs for these parameters are found to be 1.96%, 1.56%, 1.42% and 2.0%, 1.96%, 1.82% for HQ, CC, and NT respectively, which confirm the steadiness of their current responses. These results suggest that the method is robust and can provide reliable and consistent results even with minor variations in the experimental conditions.

### Simultaneous determination of HQ, CC, and NT in real water sample

**Figure 6:**
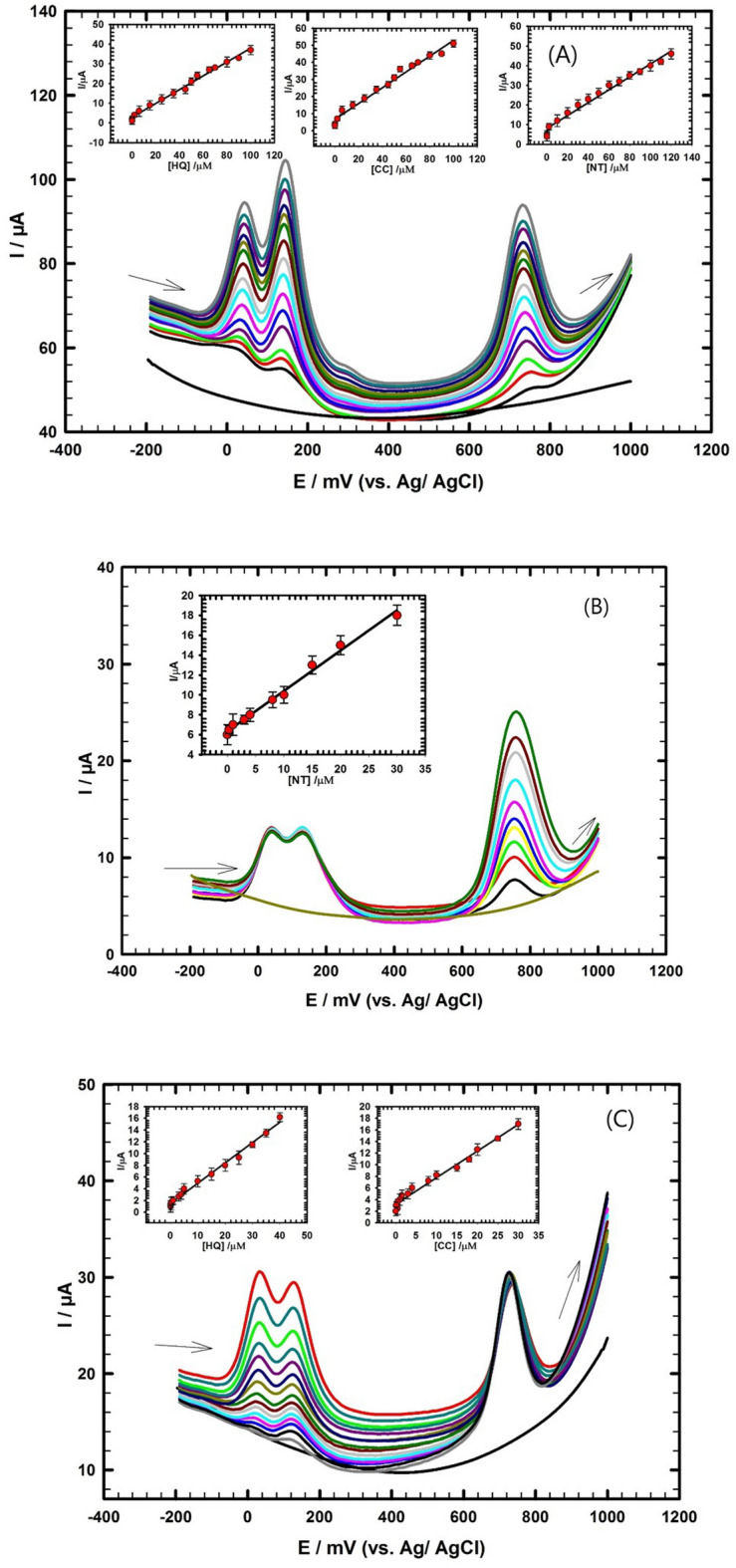
(**A**) DPVs for simultaneous determination of HQ, CC and NT in concentration ranges (0.04 → 100 µM), (0.01 → 100 µM) and (0.05 → 120 µM) in wastewater/0.1 M PBS pH 7.0; insets: the corresponding calibration curves for HQ, CC and NT using GC/PEDOT/CNT-GRO electrode. (**B**) DPVs of NT in the concentration range from (0.01 μM to 30 μM) in presence of constant concentration 20 μM of HQ and 10 µM of CC in wastewater/0.1 M PBS pH 7.0; inset: the corresponding calibration curve for NT. (**C**) DPVs of HQ and CC in the concentration ranges from (0.06 μM to 40 μM) and (0.02 μM to 30 μM) in presence of constant concentration 60 μM of NT in wastewater/0.1 M PBS pH 7.0; insets: the corresponding calibration curves for HQ and CC.

The sensor's performance is further assessed using the DPV technique to determine HQ, CC, and NT simultaneously in wastewater sample under optimized conditions. The proposed method is validated according to ICH guidelines^76^. Pulse voltammetric methods, such as DPV, are effective and rapid electroanalytical techniques with well-established advantages, including good discrimination against background current and low detection limits. The following are the parameters for the DPV experiments: E_i_ – 200 mV, E_f_ **+** 1000 mV, scan rate 20 mV/s, pulse width 50 ms, pulse period 200 ms, and pulse amplitude 10 mV. The HQ, CC, and NT are successfully detected simultaneously in wastewater sample with changing their concentrations as shown in Fig. 6A. Three distinct oxidation peaks appeared on the DPV, as the concentrations of HQ, CC, and NT increased in the ranges (0.04 → 100 µM), (0.01 → 100 µM) and (0.05 → 120 µM), respectively, their oxidation peak currents increased accordingly. Figure 6A; insets show linear fit curves between the oxidation peak current and the concentration for HQ, CC, and NT. The curves demonstrate that within the concentration range for each compound the peak currents increase linearly with their corresponding concentrations. The linear fitting equations between the oxidation peak current and the concentration for HQ, CC, and NT are as follows:

$$\begin{aligned} {\text{I}}_{{\text{p}}} (\upmu {\text{A}}) & = 0.{35}0{\text{C}}_{{{\text{HQ}}}} \left( {\upmu {\text{M}}} \right) \, + {2}.{92}, \, ({\text{R}}^{{2}} = 0.{991}) \\ {\text{I}}_{{\text{p}}} (\upmu {\text{A}}) & = 0.{\text{462C}}_{{{\text{CC}}}} \left( {\upmu {\text{M}}} \right) \, + {6}.{72}, \, ({\text{R}}^{{2}} = 0.{989}) \\ {\text{I}}_{{\text{p}}} (\upmu {\text{A}}) & = 0.{\text{331C}}_{{{\text{NT}}}} \left( {\upmu {\text{M}}} \right) \, + {7}.{71}, \, ({\text{R}}^{{2}} = 0.{997}) \\ \end{aligned}$$

The detection limits (DLs) are 8.5 nM, 3.8 nM and 6.1 nM, respectively. DLs are determined according to (S/N = 3). The GC/PEDOT/CNT-GRO sensor provides reasonable linear ranges for HQ, CC, and NT detection with DLs lower than other cited modified electrodes in previous work^17,69,77–80^ as illustrated in Supplement Table 3. The results indicate that the developed sensing method is appropriate for detecting HQ, CC, and NT simultaneously in wastewater sample without any cross-interference.

### Investigating the intermolecular effect among HQ, CC, and NT

To investigate the intermolecular effect among HQ, CC, and NT, two experiments are conducted under optimized conditions. In the first experiment, the concentration of one analyte is changed while keeping the concentration of other analytes constant, and in the second experiment the reverse is made. Firstly, the concentration of NT is changed from 0.01 to 30 µM while the concentrations of HQ and CC are fixed at (20 µM) and (10 µM), respectively, Fig. 6B. Linear relationship is obtained between the peak current and the concentration of NT as shown in (Fig. 6B; inset) with the following regression equation:

$${\text{I}}_{{\text{p}}} (\upmu {\text{A}}) = \, 0.{\text{345 C}}_{{{\text{NT}}}} \left( {\upmu {\text{M}}} \right) \, + {6}.{35},\;\;\;\; \, \left( {{\text{R}}^{{2}} = 0.{995}} \right)$$

The DL for NT is 2.4 nM.

In the second experiment, the concentrations of HQ and CC are changed from 0.06 to 40 µM and from 0.02 to 30 µM, respectively, while the concentration of NT is fixed at 60 µM, Fig. 6C. Linear relationships are obtained between the peak current and the concentration for CC and for HQ as shown in (Fig. 6C; insets) with the following regression equations:

$${\text{I}}_{{\text{p}}} (\upmu {\text{A}}) = 0.{\text{346C}}_{{{\text{HQ}}}} \left( {\upmu {\text{M}}} \right) \, + {1}.{47}, \, \;\;\;\;({\text{R}}^{{2}} = 0.{992})$$

$${\text{I}}_{{\text{p}}} (\upmu {\text{A}}) = 0.{\text{453C}}_{{{\text{CC}}}} \left( {\upmu {\text{M}}} \right) \, + {3}.{23}, \, \;\;\;\;({\text{R}}^{{2}} = 0.{989})$$

The DLs are 2.96 nM, 1.4 nM for HQ and CC, respectively.

The results show that the GC/PEDOT/CNT-GRO sensor can successfully detect the ternary mixture of HQ, CC, and NT simultaneously in their mixture without intermolecular interference. Therefore, this sensor is suitable for accurate and reliable detection of these three pollutant species.

### Selectivity

**Figure 7:**
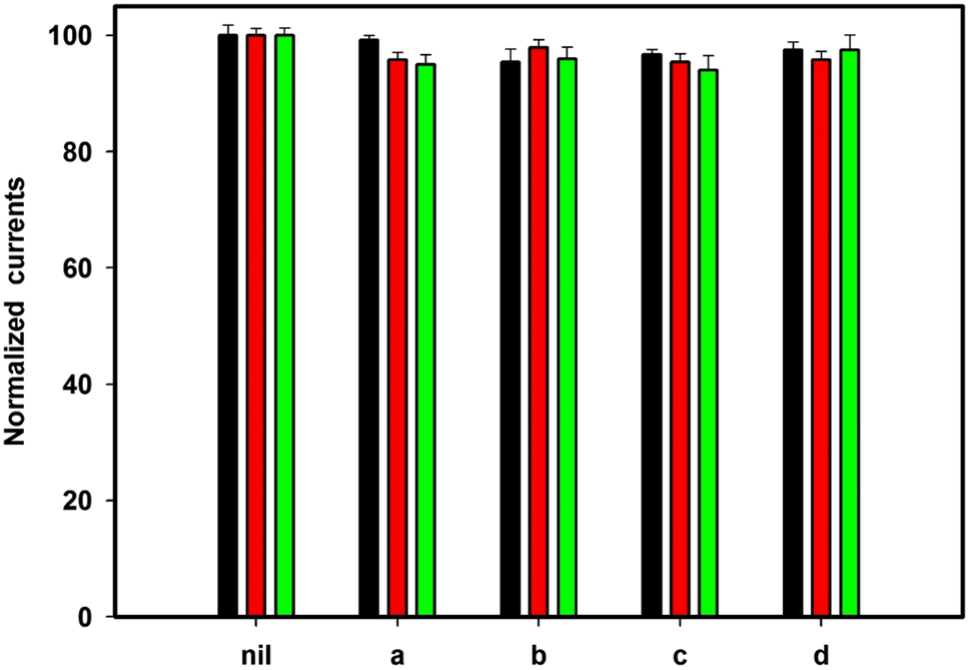
The interference study for HQ, CC and NT at GC/PEDOT/CNT-GRO in presence of 100-fold excess of interfering ions (a) Na^+^, K^+^, Mg^2+^, Cl^−^, PO_4_^3−^ (b) Pb^2+^, Ca^2+^, Cu^2+^, Cd^2+^, Mn^2+^, SO_4_^2−^, Cl^−^ and in presence of 50-fold excess of interfering organic compounds (c) 3-aminophenol, phenol, (d) aniline, citric acid; (Nil, 100 μM).

The selectivity of the sensor plays a crucial role in its practical application. Therefore, the selectivity of the modified electrode is evaluated in the presence of potentially interfering species. The ability to detect HQ, CC, and NT in real water samples in presence of various cations and anions such as K^+^, Mg^2+^, Cu^2+^, Na^+^, Cd^2+^, Pb^2+^, Mn^2+^, Ca^2+^, SO_4_^2−^, Cl^−^, and PO_4_^3−^ with 100-fold concentration has been investigated using the GC/PEDOT/CNT-GRO electrode. No significant variation of the anodic peak current (less than 4.55%) and the oxidation potential values of HQ, CC, and NT in presence of these interfering ions. Also, the current responses of the sensor show less than 5.56% variation in presence of 50-fold concentration of interfering compounds such as 3-aminophenol, aniline, phenol, and citric acid, Fig. 7. These results demonstrate the anti-interference capability of the proposed surface and a good selectivity for simultaneous detection of these pollutant species.

### Recovery

We studied the practical feasibility of the proposed method for determination of HQ, CC, and NT in wastewater sample by standard additions using the GC/PEDOT/CNT-GRO sensor. The DPV current responses of HQ, CC, and NT in wastewater sample are measured after each addition of known concentrations of HQ, CC, and NT. Each run is repeated three times after each addition and the average current responses for HQ, CC and NT are calculated. The results of the calculated concentrations and the recoveries are presented in Table 2. The recovery for HQ, CC and NT ranged from 97.7 to 101.2%, 98.9 to 102.4% and 98.1 to 102.3% respectively, indicating that the GC/PEDOT/CNT-GRO sensor has excellent recovery rate for HQ, CC, and NT determination in wastewater sample. These findings demonstrate that the GC/PEDOT/CNT-GRO electrode is an effective, reliable, and accurate for detecting these pollutants in wastewater samples.

**Table 2:** Evaluation of the accuracy and precision of the proposed method for determination of HQ, CC, and NT in wastewater sample.

Studied compounds	Concentration added (µM)	Concentration found (µM)	Recovery (%)	Standard deviation	Standard error	Confidence level	RSD (%)
HQ	0.1	0.098	97.72	0.068	0.039	0.168	2.398
	2	2.024	101.22	0.018	0.010	0.044	0.507
	15	15.177	101.18	0.117	0.068	0.290	1.438
	25	25.070	100.28	0.144	0.083	0.357	1.237
	35	35.015	100.04	0.085	0.049	0.212	0.564
CC	0.1	0.101	101.47	0.185	0.107	0.458	2.887
	2	2.048	102.38	0.144	0.083	0.356	1.962
	6	6.037	100.62	0.176	0.101	0.436	1.910
	45	44.641	99.20	0.553	0.319	1.371	2.018
	55	54.384	98.88	0.554	0.320	1.373	1.731
NT	0.1	0.099	98.75	0.193	0.112	0.480	2.662
	2	1.962	98.11	0.030	0.017	0.074	0.376
	20	20.450	102.25	0.126	0.073	0.313	0.889
	30	30.529	101.76	0.089	0.052	0.222	0.507
	40	40.305	100.76	0.026	0.015	0.065	0.125

## Conclusions

A novel method is developed for simultaneous detection of three environmental pollutants, namely HQ, CC, and NT using GC/PEDOT/CNT-GRO electrochemical sensor in real water samples. The modification of the GC electrode with PEDOT/CNT-GRO composite resulted in improvement of the electronic transport rate between the studied pollutant species and the modified composite surface. The sensor detects the HQ, CC, and NT in wide concentration ranges (0.04 → 100 µM), (0.01 → 100 µM) and (0.05 → 120 µM) with low detection limits of 8.5 nM, 3.8 nM and 6.1 nM, respectively compared to other working electrodes reported in the literature. The synergistic effect between the modifiers GRO, CNTs, and PEDOT increases the electro-catalytic activity of the PEDOT/CNT-GRO composite toward the oxidation of HQ, CC, and NT. Besides, the advantageous structural, morphological, conducting, and other specific properties of the nanocomposite improve the voltametric separation between the HQ, and CC isomers that oxidize at nearly similar potentials at conventional electrodes. Good potential peak separations: 117 mV and 585 mV are obtained between the HQ-CC, and CC-NT. Further, the sensor has perfect stability, and reproducibility, good anti-interference capability and excellent selectivity for the simultaneous determination of HQ, CC, and NT in presence of common interfering ions and compounds in wastewater samples, making it a reliable tool for detecting these pollutant species in complex environmental samples. Thus, the sensor has a good impact for actual practical applications.

### Supplementary Information

Supplementary Information.

## Data Availability

All data generated or analyzed during this study are included in this published article [and its supplementary information files].
