# Supplementary material for: Electrochemical sensor based on PEDOT/CNTs-graphene oxide for simultaneous determination of hazardous hydroquinone, catechol, and nitrite in real water samples
Source: Sci Rep. 2024 Mar 7;14:5654. doi: 10.1038/s41598-024-54683-9 (PMC10920748; doi:10.1038/s41598-024-54683-9)
Supplement: Supplementary file 1 — Supplementary Information. [file 41598_2024_54683_MOESM1_ESM.docx]

**Electrochemical sensor based on PEDOT/CNTs**-**graphene oxide for simultaneous determination of hazardous hydroquinone, catechol, and nitrite in real water samples**

**Yousef M. Ahmed, Mahmoud A. Eldin, Ahmed Galal, Nada F. Atta***


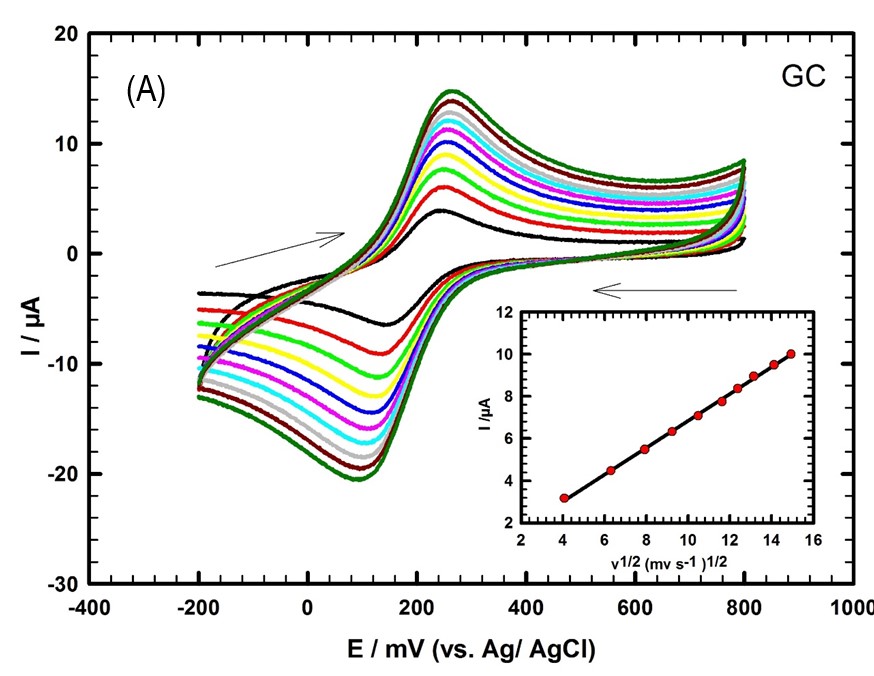


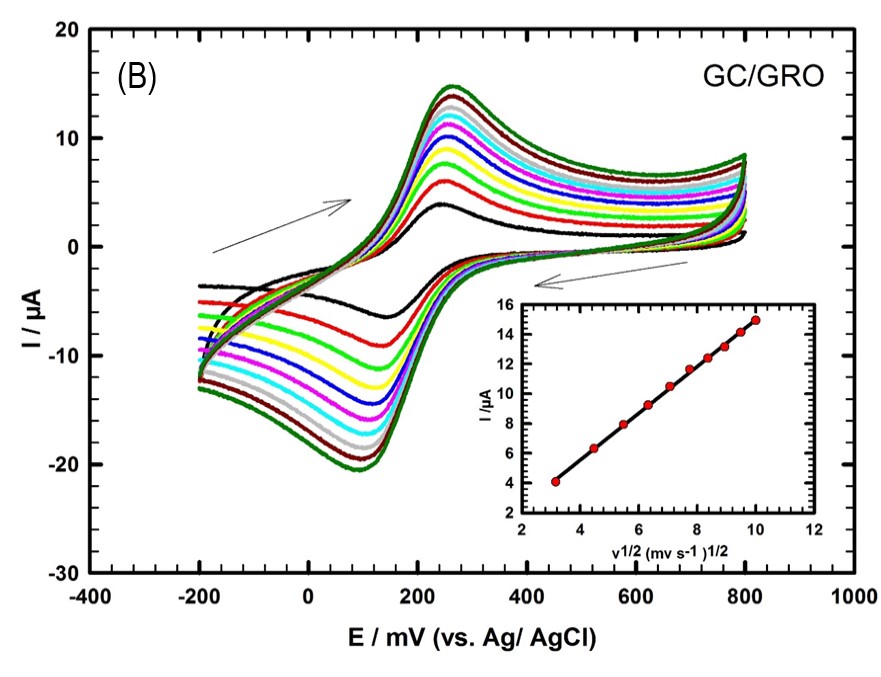


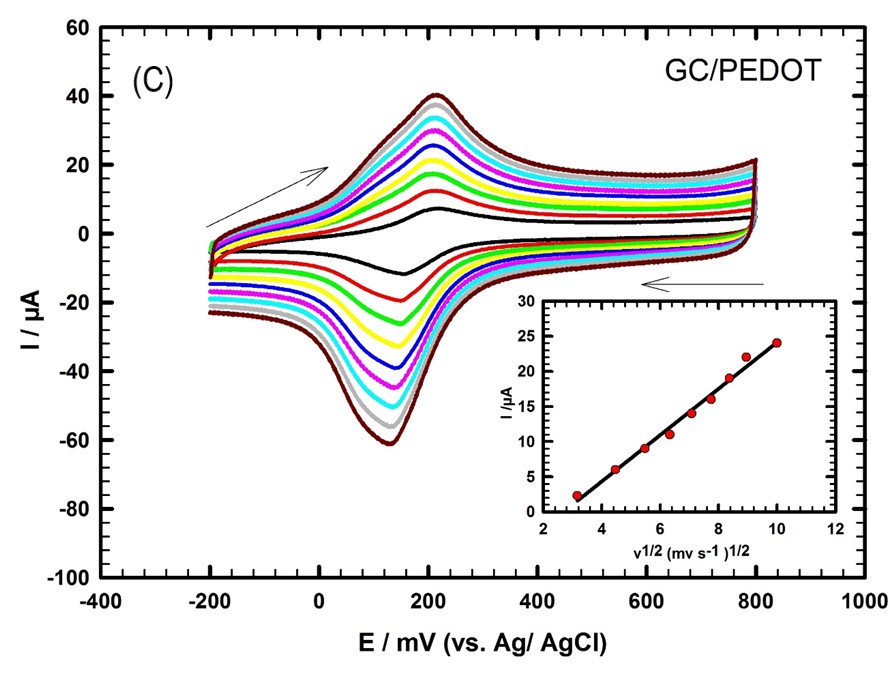


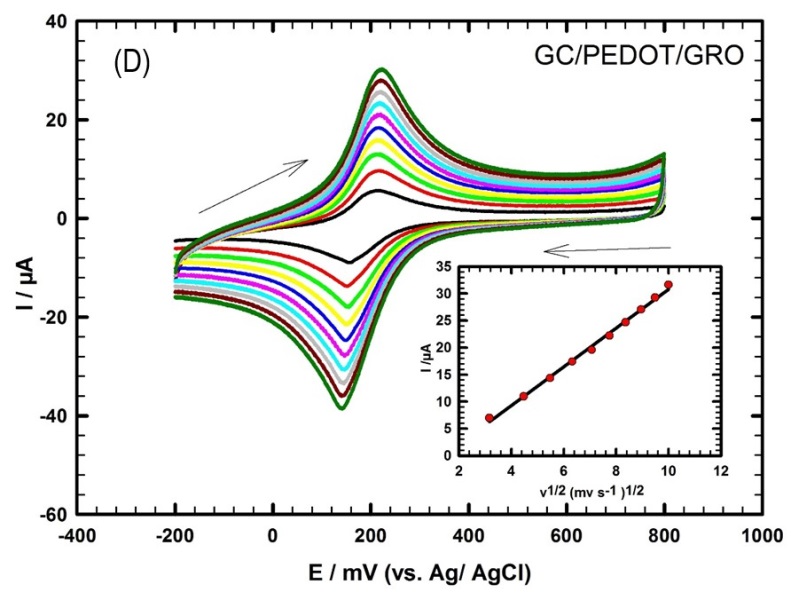


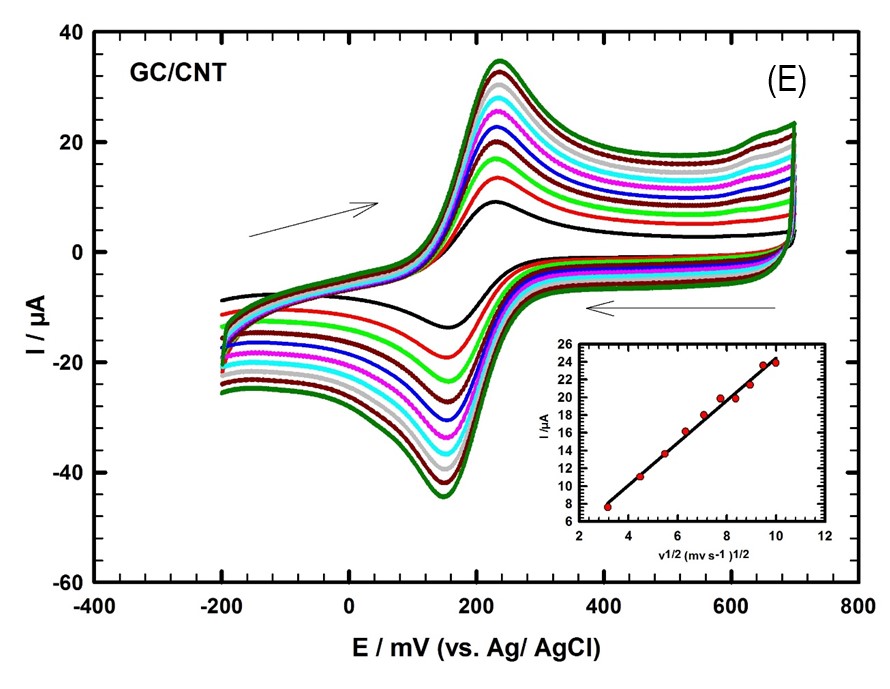


+
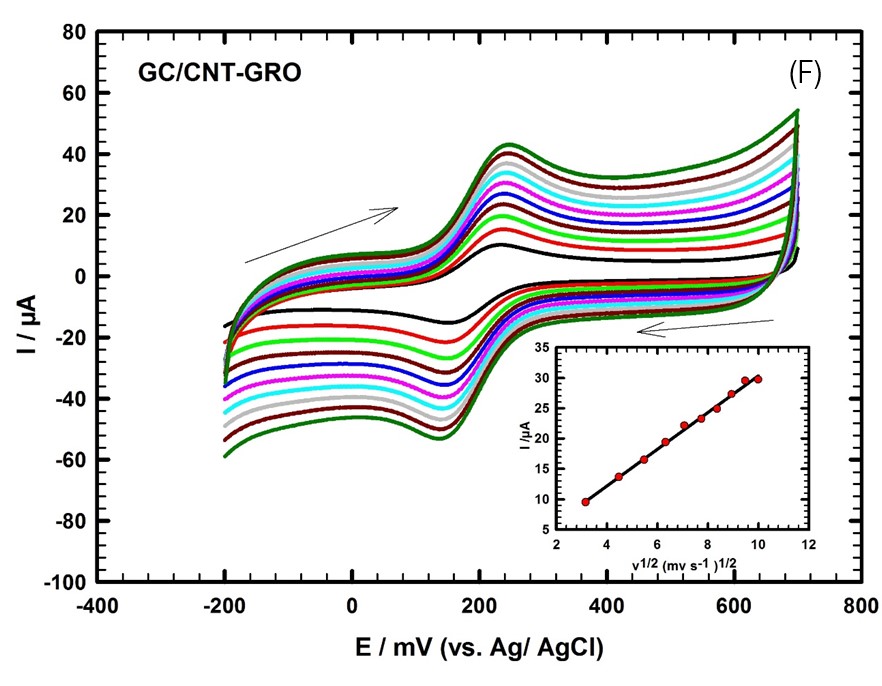


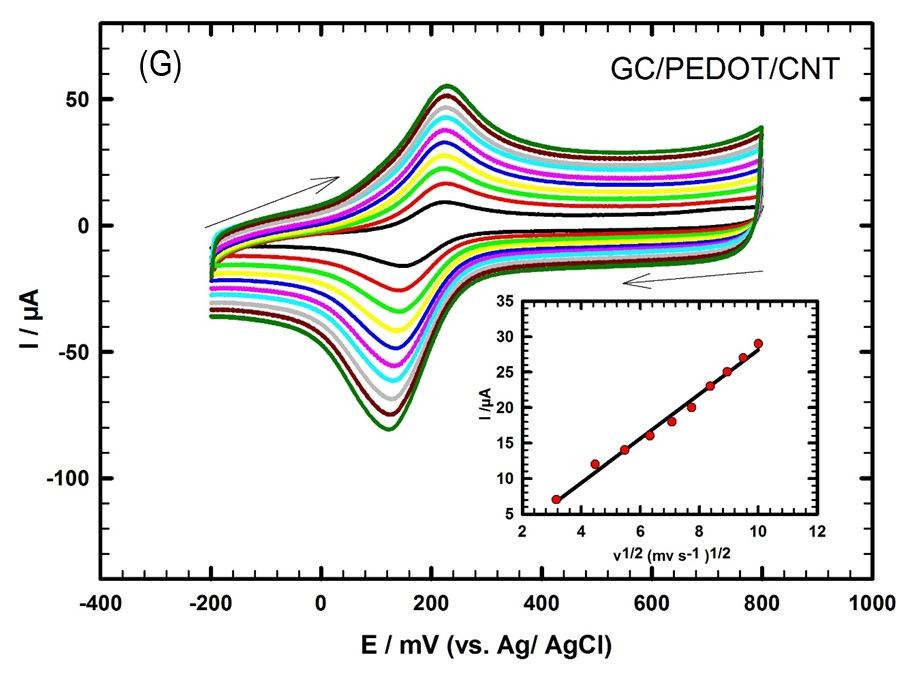


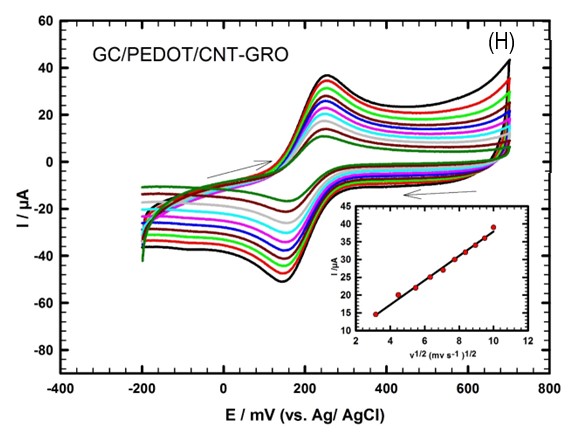


**Supplement Figure 1 (A-H):** CV experiments in 1.0 mM K_3_[Fe(CN)_6_] system using different working electrodes: bare GC, GC/RGO, GC/PEDOT, GC/PEDOT/GRO GC/CNT,  GC/CNT-GRO, GC/PEDOT/CNT, GC/PEDOT/CNT-GRO, respectively. Insets: Corresponding [relations between oxidation peak current and square root of scan rate](#_Toc136482542)

/


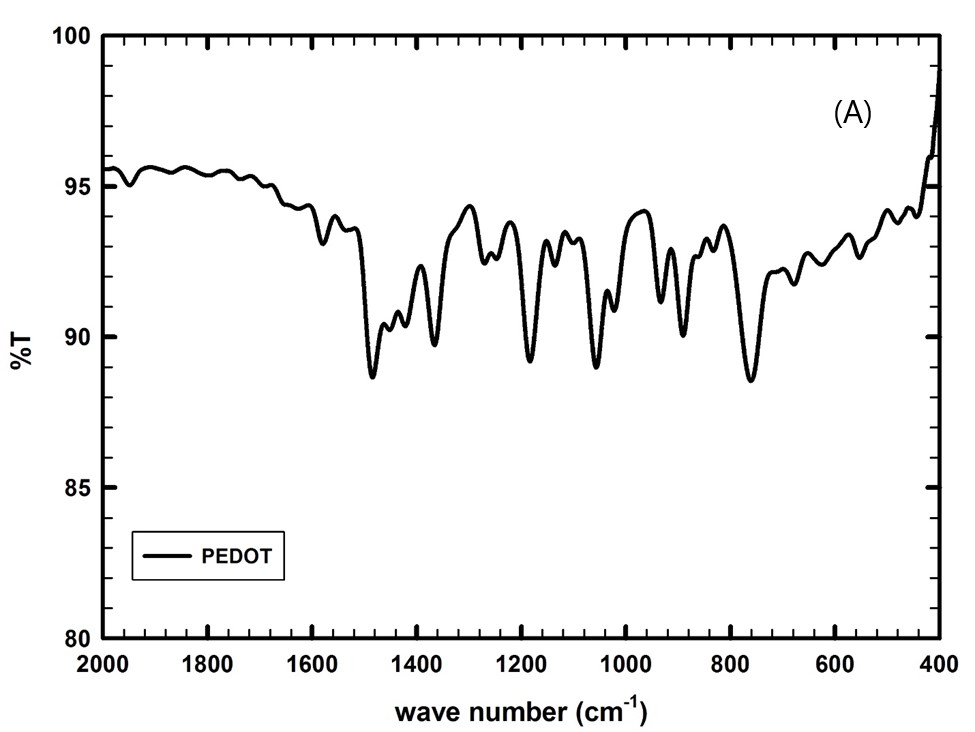


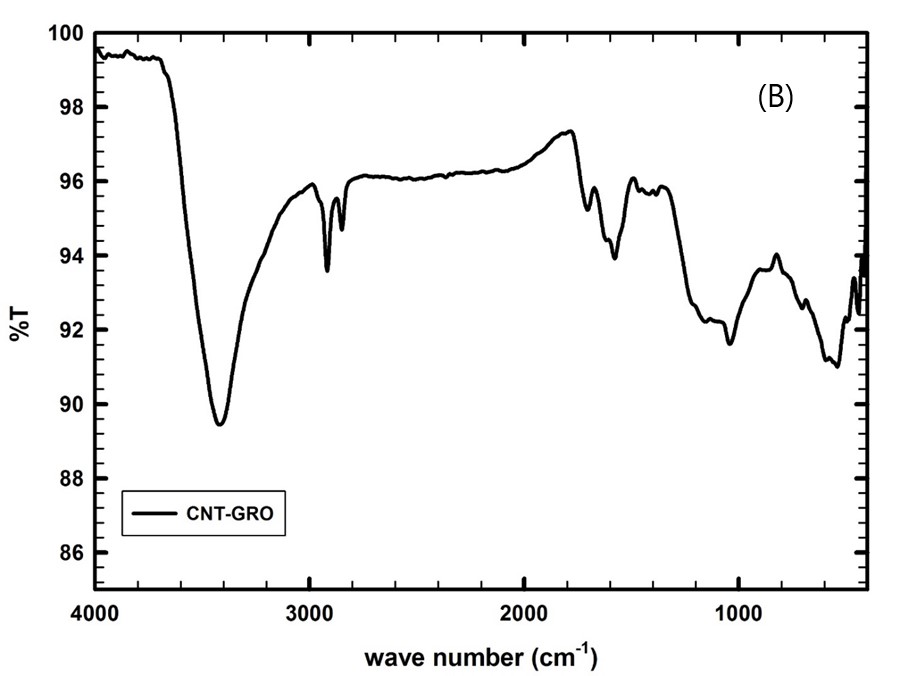


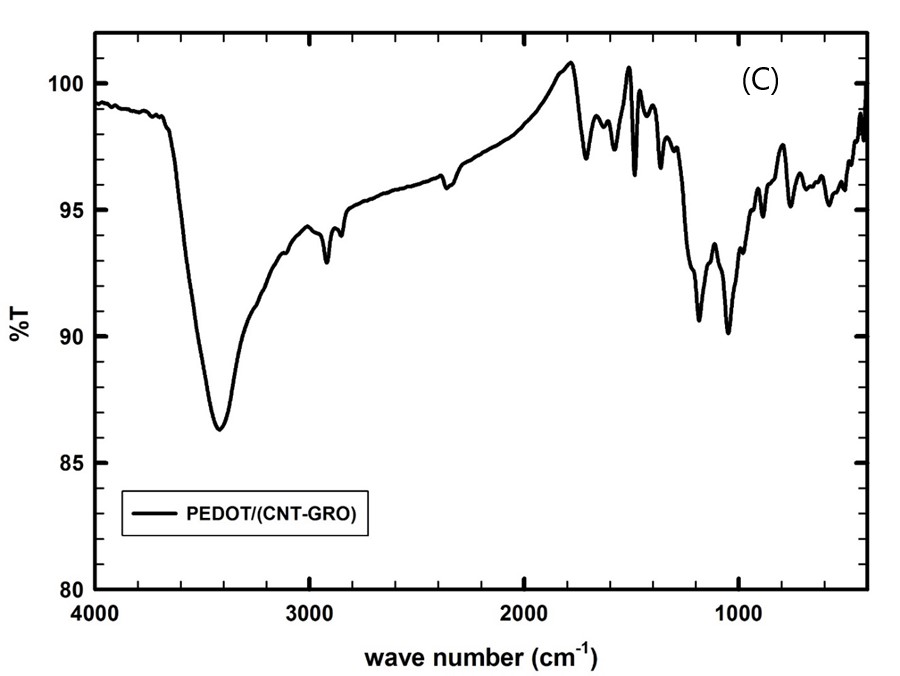


**Supplement Figure 2:** FTIR of (A) PEDOT, (B) CNT-GRO, (C) PEDOT/CNT-GRO


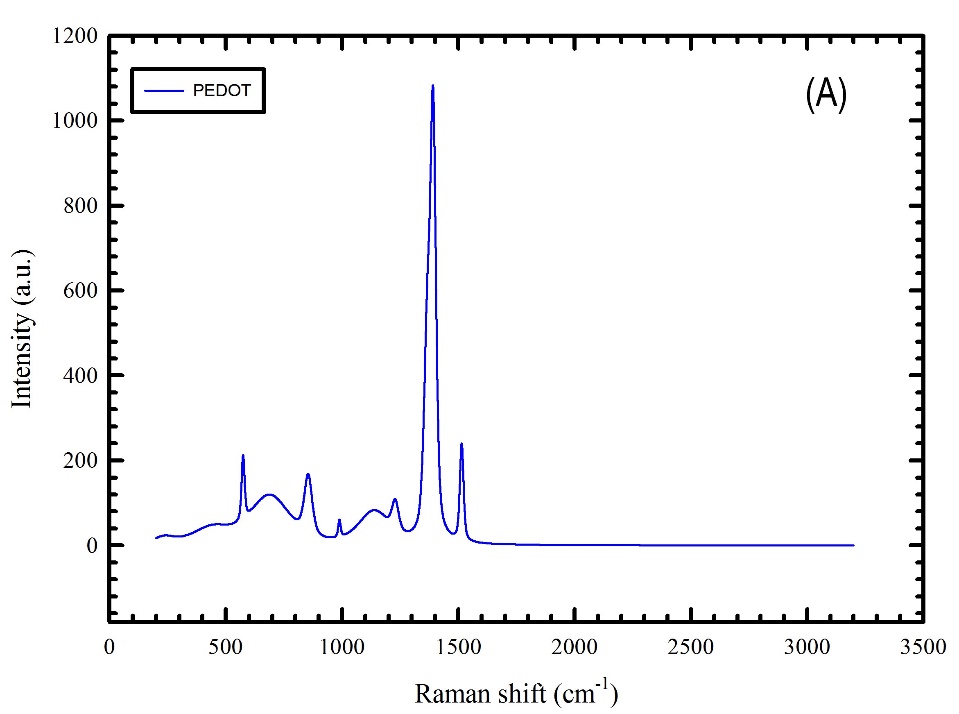


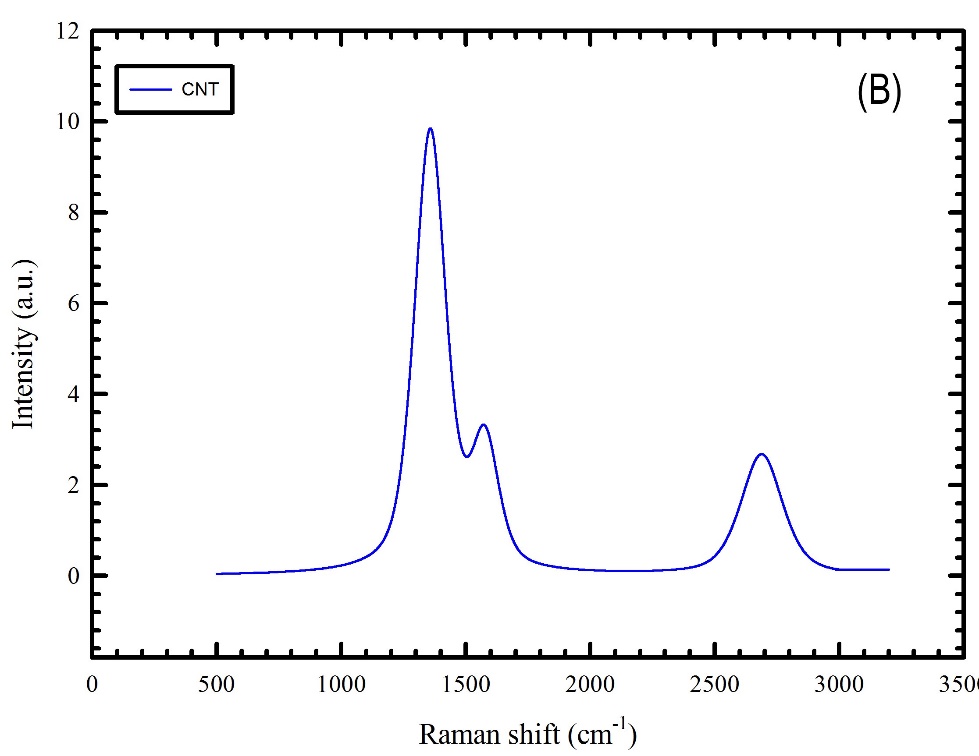


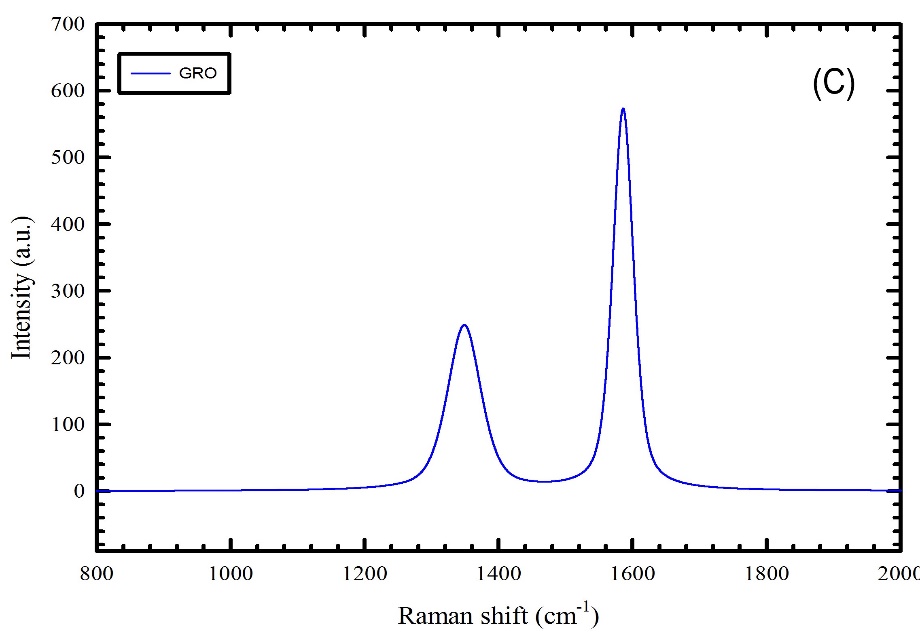


**Supplement Figure 3:** Raman of (A) GC/PEDOT, (B) GC/CNT, (C) GC/GRO


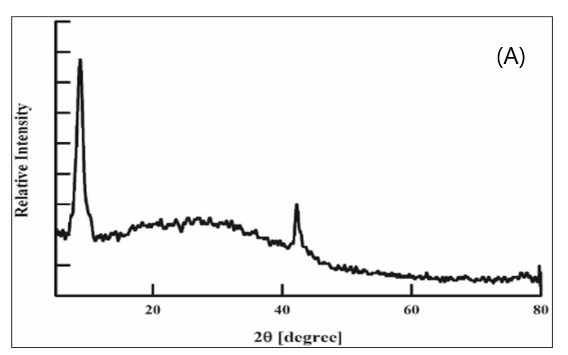


**Supplement Figure 4A:** XRD pattern of GRO prepared by Hummer's method.


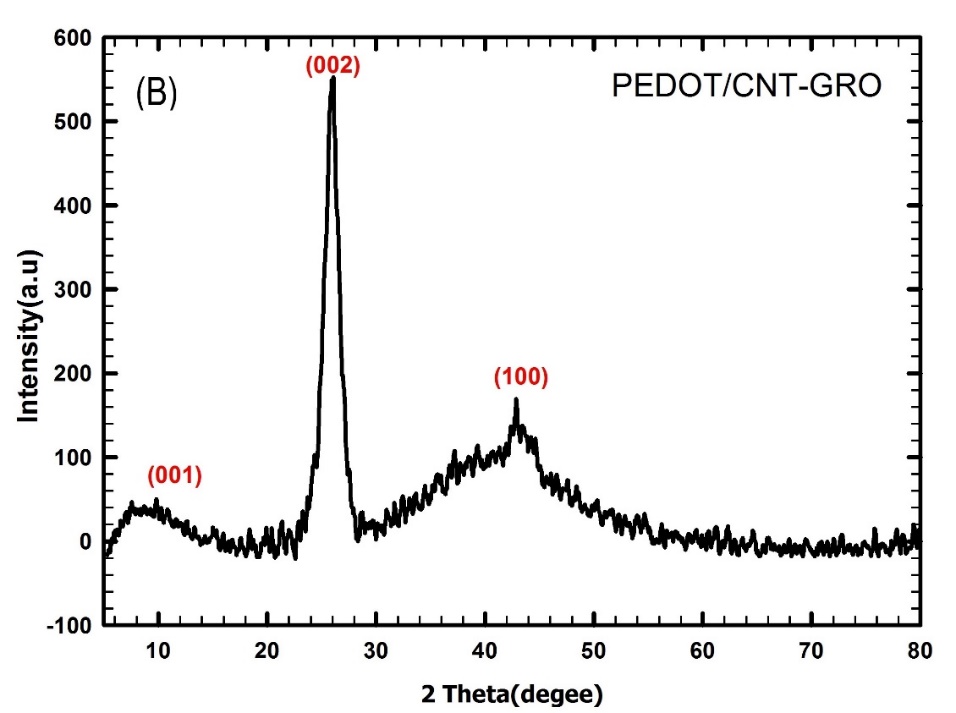


**Supplement Figure 4B:** XRD pattern of PEDOT/CNT-GRO


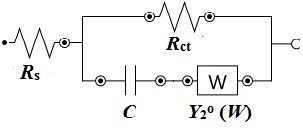


Equivalent Circuit 1


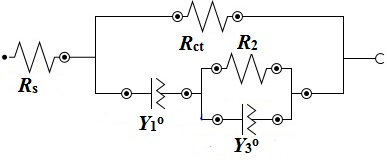


Equivalent Circuit 2


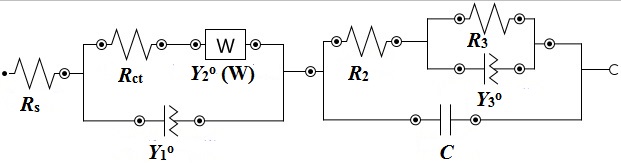


Equivalent Circuit 3

**Supplement Figure 5:** Equivalent circuits used for fitting.


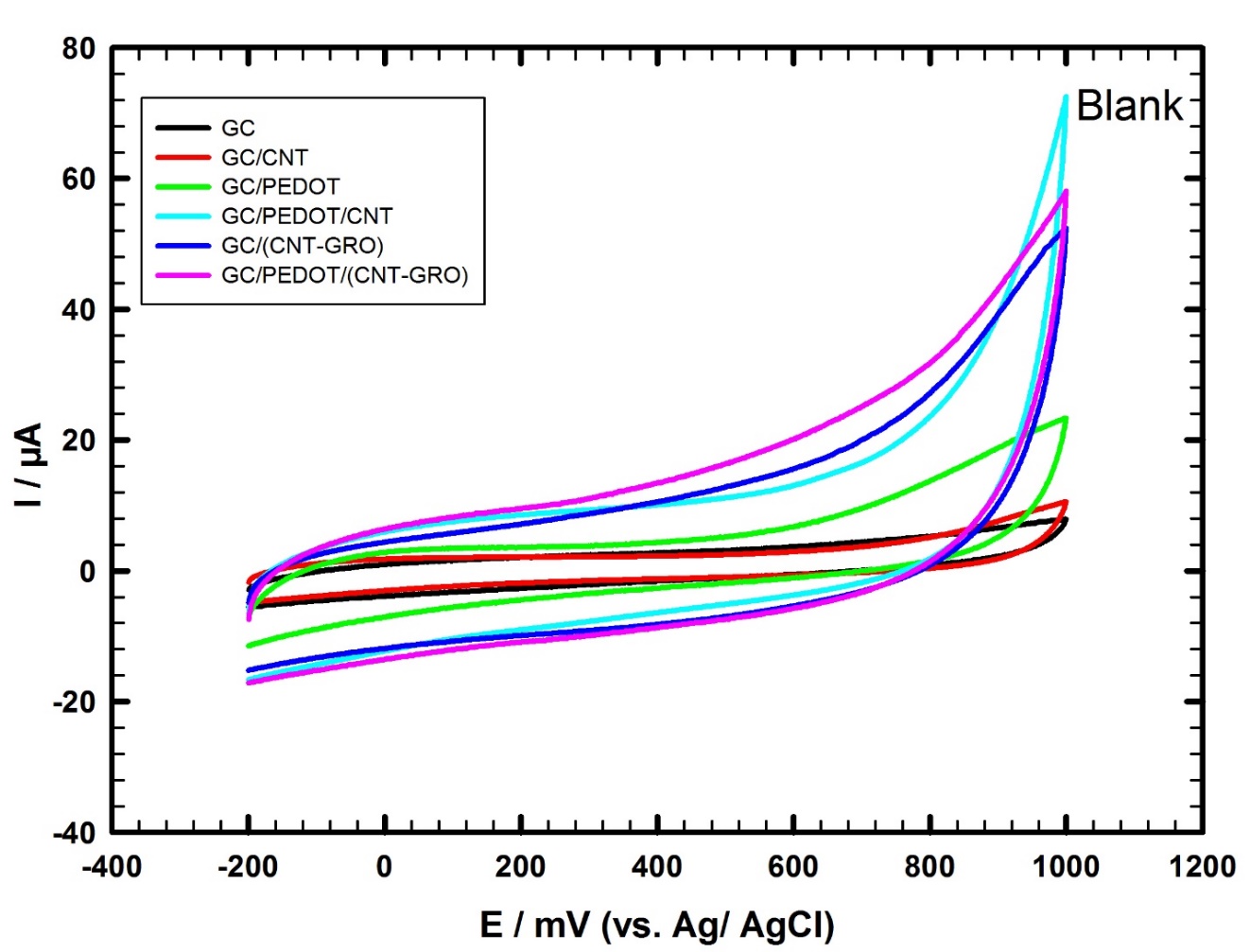


**Supplement Figure 6:** The banks for all the modified working electrodes


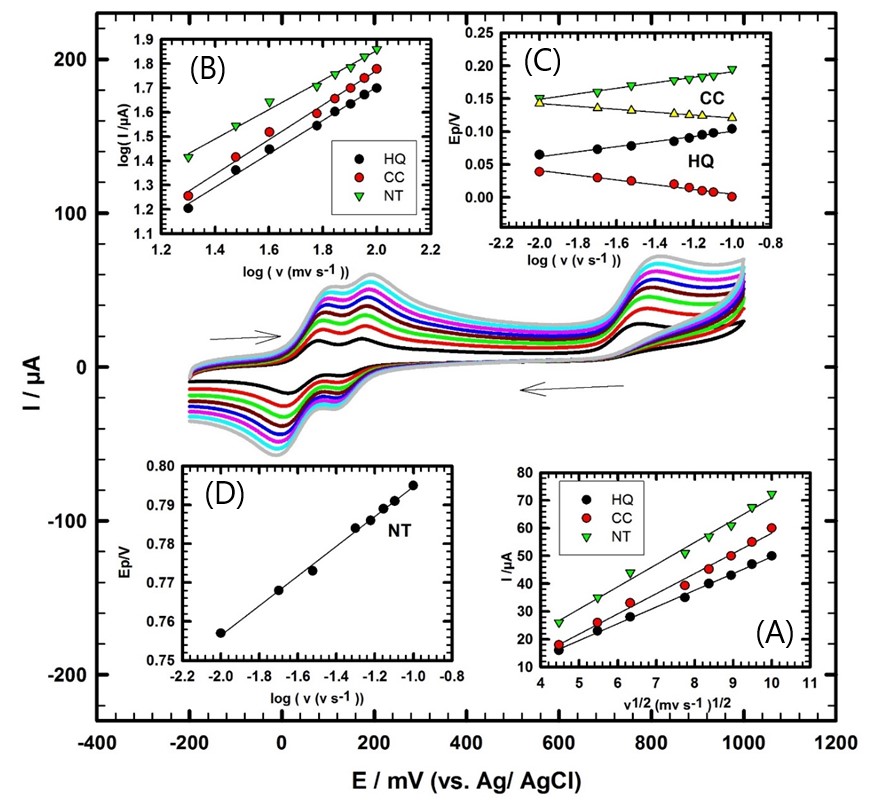


**Supplement Figure** **7:** Effect of varying the scan rate on the CVs of 100 µM HQ, 100 µM CC, and 120 µM NT/0.1 M PBS (pH 7.0); insets: (A) relation between oxidation peak current and square root of scan rate. (B) relation between log oxidation peak current and log scan rate. (C) relation between oxidation potential and log scan rate for HQ and CC. (D) relation between oxidation potential and log scan rate for NT.


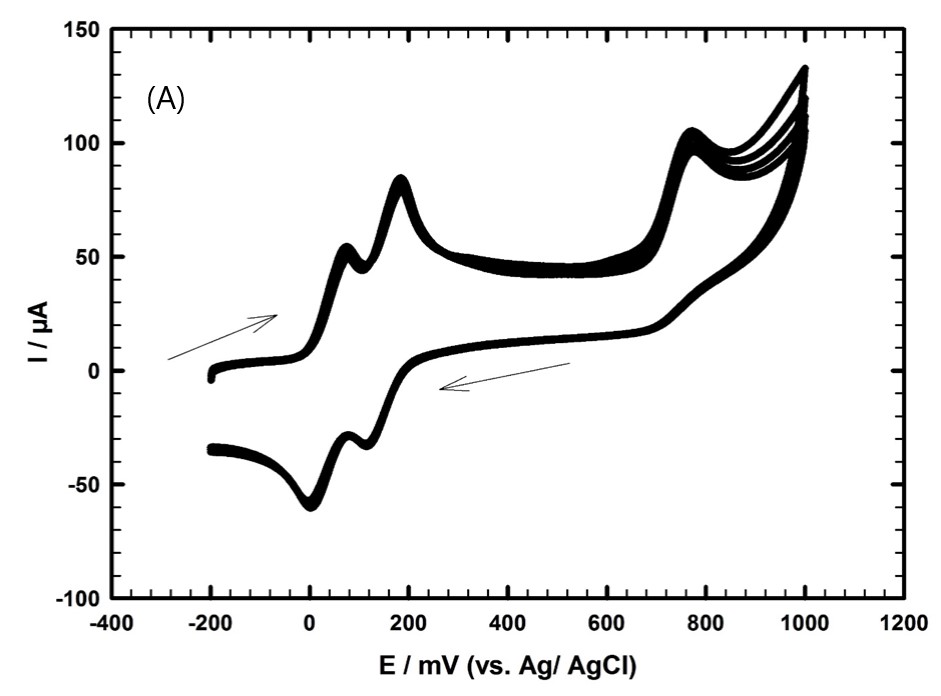


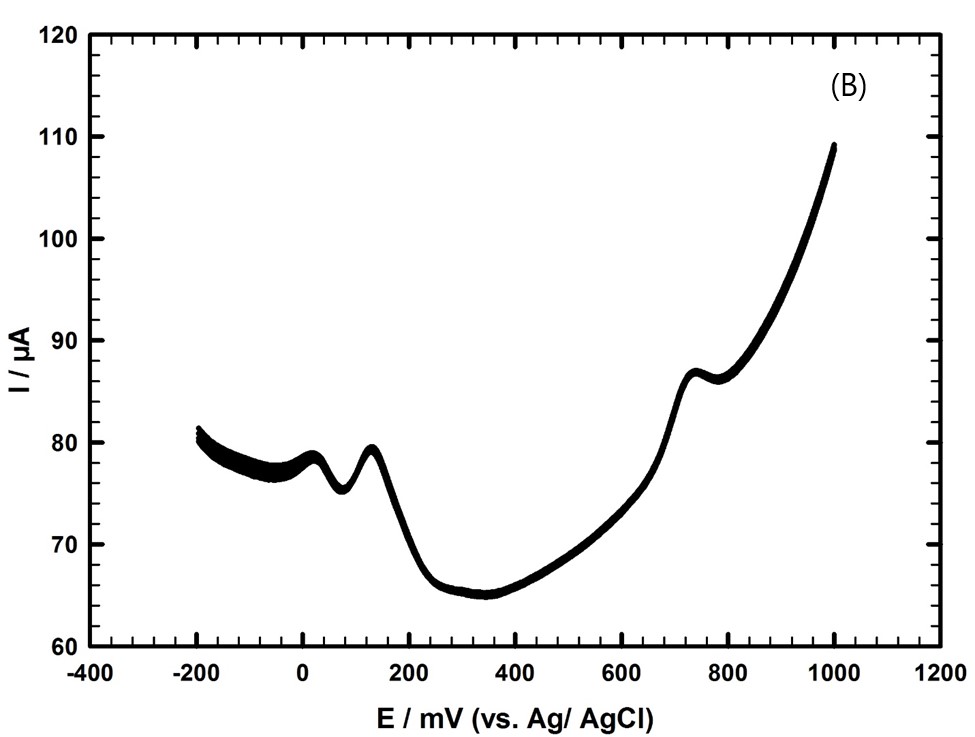


**Supplement Figure 8:** (A) Repeated CVs (25 cycles) for testing the stability using GC/PEDOT/CNT-GRO electrode in 100 µM HQ, 100 µM CC, and 120 µM NT/0.1 M PBS (pH 7.0); scan rate 50 mVs^–1^. (B) Repeated DPVs (15 cycles) for testing the stability using GC/PEDOT/CNT-GRO electrode in 2 µM HQ, 5 µM CC, and 3 µM NT/0.1 M PBS (pH 7.0).

**Supplement Table 1: The devices used in this work.**

| **Instrument** | **Uses** |
| --- | --- |
| BAS-Epsilon electrochemical equipment with a cell contained three electrodes (GCE (Ф: 3 mm), platinum wire and Ag/AgCl (3 M KCl). | Voltammetry measurements |
| Quanta 250 FEG instrument | Scanning electron microscopy FE-SEM measurement |
| Quanta 250 FEG instrument | Energy dispersive X-ray spectroscopy (EDX) measurement |
| Witec alpha 300R confocal Raman microscope with a 532 nm Nd:Yag laser. | Raman spectrum |
| Shimadzu IR-Affinity1 Spectrometer | Fourier transform infrared (FTIR) |
| X-ray diffraction (XRD) instrument D8 Advance (Bruker, Germany) spectrometer | X-ray diffraction |

**Supplement Table 2:** **summarizes the electrochemical data for the oxidation of the studied compounds.**

|  | HQ | | CC | | NT | |
| --- | --- | --- | --- | --- | --- | --- |
| Electrode | I_pa_ | E_pa_ | I_pa_ | E_pa_ | I_pa_ | E_pa_ |
|  | (µA) | (mV) | (µA) | (mV) | (µA) | (mV) |
| Bare GC | - | - | - | - | 3 | 806 |
| GC/PEDOT | 21 | 91 | 20 | 198 | 50 | 766 |
| GC/PEDOT/CNT | 40 | 83 | 31 | 195 | 76 | 791 |
| GC/PEDOT/GRO | - | - | - | - | 24 | 865 |
| GC/CNT-GRO | 27 | 41 | 28 | 145 | 32 | 745 |
| GC/PEDOT/CNT-GRO | 106 | 96 | 82 | 213 | 111 | 798 |

**Supplement Table 3:** **Comparison of GC/PEDOT/CNT-GRO with different modified electrodes mentioned in literature for HQ, CC, and NT determination.**

| Studied compounds | HQ | | CC | | NT | | Ref. |
| --- | --- | --- | --- | --- | --- | --- | --- |
| Modified electrodes | Linearity  range  (µM) | Detection limit  (µM) | Linearity  range  (µM) | Detection limit  (µM) | Linearity  range  (µM) | Detection limit  (µM) |  |
| CLS/GCE | 1–700 | 0.15 | 1–3000 | 0.11 | 0.5–4000 | 0.09 | 17 |
| MWCNT-SH@Au-GR/GPE | 54.5-1250 | 4.17 | 11.0-126 | 1 | 86.0–7500 | $\text{23.5}$ | 69 |
| PANI/ABS/GCE | - | $\text{-}$ | - | $\text{-}$ | 0.3-35 | 0.48 | 77 |
| PEDOT/GO/GCE | 2.5-200 | 1.6 | 2-400 | $\text{1.6}$ | - | $\text{-}$ | 78 |
| GO/MoS_2_/PEDOT | - | - | - | - | 1-1000 | 0.059 | 79 |
| Ni/N-GO/GCE | 1.4-800 | 0.16 | 1-800 | 0.06 | - | - | 80 |
| GC/PEDOT/CNT-GRO | 0.04-100 | 0.0085 | 0.01-100 | 0.0038 | 0.05-120 | 0.0061 | This work |

CLS: Lotus Stem derived Porous Carbon; MWCNTs: multi-wall carbon nanotubes; MWCNT-SH@Au-GR/GPE: Gold nanoparticle–graphene nanohybrid bridged 3-amino-5-mercapto-1,2,4-triazole-functionalized multiwall carbon nanotubes; PANI: polyaniline; ABS: 4-aminobenzenesulfonic diazonium salt; PEDOT: poly(3,4-ethylenedioxythiophene); GO: graphene oxide.
